# Continental-scale, data-driven predictive assessment of eliminating the vector-borne disease, lymphatic filariasis, in sub-Saharan Africa by 2020

**DOI:** 10.1186/s12916-017-0933-2

**Published:** 2017-09-27

**Authors:** Edwin Michael, Brajendra K. Singh, Benjamin K. Mayala, Morgan E. Smith, Scott Hampton, Jaroslaw Nabrzyski

**Affiliations:** 1 Department of Biological Sciences, University of Notre Dame, Galvin Life Science Center, Notre Dame, IN 46556 USA; 2 Center for Research Computing, University of Notre Dame, Notre Dame, IN 46556 USA

**Keywords:** Neglected tropical diseases, Vector-borne diseases, Lymphatic filariasis, Sub-Saharan Africa, Spatial scale, Parasite transmission heterogeneity, Hierarchical modeling, Scientific computational discovery of knowledge, Data discovery, Data-driven parasite transmission modeling, Mass drug administration, Vector control, Parasite elimination programs

## Abstract

**Background:**

There are growing demands for predicting the prospects of achieving the global elimination of neglected tropical diseases as a result of the institution of large-scale nation-wide intervention programs by the WHO-set target year of 2020. Such predictions will be uncertain due to the impacts that spatial heterogeneity and scaling effects will have on parasite transmission processes, which will introduce significant aggregation errors into any attempt aiming to predict the outcomes of interventions at the broader spatial levels relevant to policy making. We describe a modeling platform that addresses this problem of upscaling from local settings to facilitate predictions at regional levels by the discovery and use of locality-specific transmission models, and we illustrate the utility of using this approach to evaluate the prospects for eliminating the vector-borne disease, lymphatic filariasis (LF), in sub-Saharan Africa by the WHO target year of 2020 using currently applied or newly proposed intervention strategies.

**Methods and Results:**

We show how a computational platform that couples site-specific data discovery with model fitting and calibration can allow both learning of local LF transmission models and simulations of the impact of interventions that take a fuller account of the fine-scale heterogeneous transmission of this parasitic disease within endemic countries. We highlight how such a spatially hierarchical modeling tool that incorporates actual data regarding the roll-out of national drug treatment programs and spatial variability in infection patterns into the modeling process can produce more realistic predictions of timelines to LF elimination at coarse spatial scales, ranging from district to country to continental levels. Our results show that when locally applicable extinction thresholds are used, only three countries are likely to meet the goal of LF elimination by 2020 using currently applied mass drug treatments, and that switching to more intensive drug regimens, increasing the frequency of treatments, or switching to new triple drug regimens will be required if LF elimination is to be accelerated in Africa. The proportion of countries that would meet the goal of eliminating LF by 2020 may, however, reach up to 24/36 if the WHO 1% microfilaremia prevalence threshold is used and sequential mass drug deliveries are applied in countries.

**Conclusions:**

We have developed and applied a data-driven spatially hierarchical computational platform that uses the discovery of locally applicable transmission models in order to predict the prospects for eliminating the macroparasitic disease, LF, at the coarser country level in sub-Saharan Africa. We show that fine-scale spatial heterogeneity in local parasite transmission and extinction dynamics, as well as the exact nature of intervention roll-outs in countries, will impact the timelines to achieving national LF elimination on this continent.

**Electronic supplementary material:**

The online version of this article (doi:10.1186/s12916-017-0933-2) contains supplementary material, which is available to authorized users.

## Background

Recently, there has been increasing interest in assessing the prospects of currently applied and proposed nation-wide interventions for achieving the global elimination or control of the major preventable helminthic diseases, ranging from soil-transmitted helminthiases to schistosomiasis, onchocerciasis, and lymphatic filariasis (LF) [1–5]. Partly, this is in response to the urgent policy demands for more accurate scientific information for determining if the roadmap set by the World Health Organization (WHO), based on sustaining and expanding drug access programs, will accomplish the elimination or control of these neglected tropical diseases (NTDs) by the target year of 2020 [6]. In part, this interest also reflects the recent advances made in the areas of data science and computational epidemiology that increasingly enable the parameterization and execution of complex mechanistic models for simulating the outcomes of interventions reliably over large spatial domains [7–11].

Previous modeling studies aiming to evaluate the prospects of meeting the goals of NTD programs at large regional or country scales have mainly employed two basic approaches. First, generalized parasite transmission models relying on parameter values obtained from limited datasets have been used to simulate intervention outcomes for a range of input values [1, 2, 12]. In essence, this approach assumes that employing models parameterized at point-support spatial scales, i.e., using parameters and model structures originally defined from data collected at a few spatial sites, invariantly across a region is valid for mimicking regional-scale parasite population dynamics [13–15]. More recently, approaches that use global grid-based parameter search methods for calibrating transmission models against either mean national-level infection values or a range of subgrid values within countries have been applied for undertaking these modeling investigations [1–5]. While these studies have provided important strategic insights into the impacts of intervention options on likely timelines to parasite elimination, an implicit assumption behind these methods is stability and stationarity in the fine-scale pattern-process relationships used to develop the transmission models [13, 14, 16–18]. If spatial nonstationarity or heterogeneity occurs in these pattern-process relationships, then significant aggregation errors can occur, severely biasing the model predictions produced and used at broader or coarser spatial levels [16–18]. Such predictions will significantly underestimate the full range of heterogeneity in infection dynamics and consequently the outcomes of interventions across a spatial domain [19].

We have previously shown how a key variable connected with LF elimination, viz., infection breakpoints or thresholds, is highly sensitive to local transmission conditions, and how this heterogeneity in the values of this variable can play a significant role in generating between-site variability in the timelines to parasite elimination as a result of applying interventions across a spatially heterogeneous domain [20–22]. This outcome indicates the crucial need to address spatial heterogeneities in LF transmission dynamics if better predictions of the interventions proposed to eliminate this parasitic disease are to be delivered. It also implies that modeling frameworks that do not address such heterogeneity will not be able to offer the explicit predictions across space required by policy makers desiring to understand where programs are working and where they are unlikely to meet goals so that tactically targeted remedial actions focused on these aberrant sites can be applied. These considerations imply that to support undertaking better model-based LF control decision analysis at large regional or global scales, development and use of prediction platforms that can address the full range as well as uncertainty in the expected system response across the entire spatial domain of interest will be of paramount importance [13].

Learning parasite transmission models that take a fuller account of heterogeneous dynamics across a spatial domain is a difficult task, but the increasing availability of geolocated demographic, intervention, and disease data [23–26] together with growing advances made in computational science approaches to knowledge discovery, particularly in the areas of (1) high performance grid-based computing and programming [8, 11], (2) data discovery, integration, and assembly [11, 19, 27–31], and (3) data-driven approaches for inferring models from measurements [32–38], mean that simulating disease dynamics and responses to interventions effectively across heterogeneous spatially structured environments at large scales are now becoming increasingly feasible. Bayesian data-driven modeling frameworks have received considerable attention in this regard given their ability for not only facilitating the induction of a dynamical system from data, but also in the use of multiple data sources for constraining the parameters of a model to capture the local transmission features of a spatial setting [21, 22, 33, 39–41].

In this paper, we describe the development of a spatially hierarchical data-driven computational platform to serve as a tool for supporting the simulation of the heterogeneous transmission dynamics and control of the major vector-borne macroparasitic disease, lymphatic filariasis, across a major endemic spatial domain, focusing here on the sub-Saharan African continent. We begin by describing how such a platform can allow estimation of the local population dynamics of this disease across this spatially complex disease-endemic continent by facilitating the learning of locality-specific transmission models from georeferenced data. We then use the discovered local models in conjunction with LF intervention data assembled for each relevant endemic country to highlight how such a system can be used to investigate the emergent policy questions germane to the elimination of this highly debilitating disease from this important endemic continent, viz., (1) which countries are on course to meet the LF elimination target year of 2020, (2) which are unlikely to meet this goal, and (3) which remedial strategies are best suited to enhance disruption of parasite transmission most effectively in the latter case. We also contrast the findings with those resulting from recently conducted national-level intervention modeling work [1–5, 12] by focusing on two themes: (1) the importance of constraining model parameters to reflect the complexity of subgrid transmission heterogeneities with multiple localized input data and (2) the need for addressing such heterogeneous transmission dynamics for minimizing aggregation error when making coarse-scale predictions.

## Methods

### Overview of modeling framework

Our proposed approach for addressing the problem of dealing with spatial heterogeneities in LF transmission and extinction dynamics for generating predictions of the effects of interventions at the required aggregated policy levels ranging from district to national scales is essentially hierarchical in nature. In particular, it is based on three coupled components: (1) methods to facilitate assembly/discovery of the relevant model input data at local settings, (2) a data-driven modeling method that can use such data for identifying locality-specific LF models, and (3) an algorithm for combining the heterogeneous simulations from local settings for making upscaled regional predictions. Below, we begin by describing the methods used to execute each of the three components above, and follow this by outlining the scientific workflow we used to implement this hierarchical modeling approach for performing this study.

### LF data assembly

Developing site-specific parasite transmission models crucially requires input data, viz*.*, exogenous forcing variables and initial state variables, defining a site to facilitate constraining a model’s parameter space so that the effects of local transmission conditions are captured reliably [21, 22, 42, 43]. For modeling impacts of interventions, the type of control carried out in a site together with data on frequency, coverage, and duration are also similarly required. A challenge concerns the availability of all of these data at every site selected in the modeling process; if these data are not available everywhere, then it raises the question of how best to learn about the model inputs for every required location from the available sparse data [33]. These data, when available, are normally also contained within multiple databases maintained by various data providers, with each dataset having unique data access protocols, file formats, and semantics [27, 29, 30]. This heterogeneity and the gaps in source data mean that to derive the input data needed to develop and run site-specific LF models, informatics and analytical tools that can combine empirical data discovery with data integration and estimation protocols will also be required [10, 33, 39]. Below, we describe the combination of data discovery and estimation methods we followed to assemble the site-specific data needed to carry out our landscape-wide modeling exercise under each type/category of input data.

### Baseline microfilaremia (mf) age prevalence

**Fig. 1:**
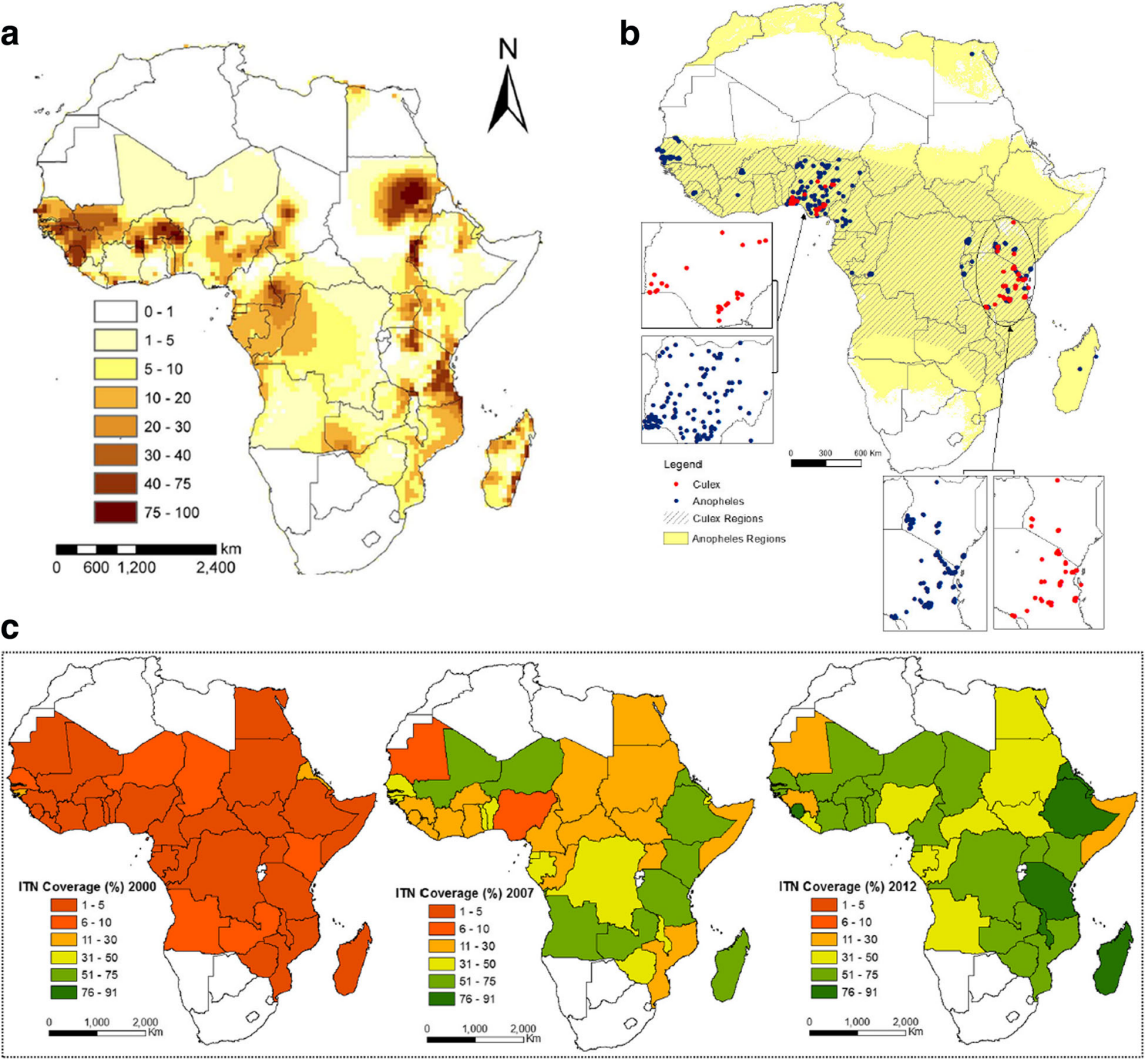
Mapped inputs for modeling the baseline transmission dynamics and effects of interventions against lymphatic filariasis (LF) in sub-Saharan Africa. **a** A smooth map of the estimates of LF prevalence is shown. The map was created using a multivariate Bayesian generalized linear spatial model, as described in [44]. **b** Smooth maps of the annual biting rates (ABRs) of *Anopheles* and *Culex* mosquitoes were created by simple kriging of ABR data obtained from literature searches and public databases (e.g., the Malaria Atlas Project (MAP)/Malaria Risk in Africa (MARA) databases). The observed data points are also shown. The *Culex* distribution is patchier than that of *Anopheles*, so we consider the *Anopheles* model to apply wherever *Anopheles* mosquitoes are implicated in transmission (Table 1). Only in those areas where *Anopheles* mosquitoes are not implicated at all did we use the *Culex* model. Note, however, that given the sparseness of the ABR data as shown on the map, we used model-estimated ABR values in the modeling exercise described in the text (see Methods). **c** Country-level coverages of bed nets (i.e., insecticide-treated nets (ITNs) interpolated from Admin1 data. Smoothed annual maps were developed for 2000–2012; here we show data for years 2000, 2007, and 2012

We used a unique LF database containing mf prevalence data for a large number of endemic settings in Africa (a total of 664 data points assembled from the published and gray literatures), which we had previously compiled for use with a Bayesian geostatistical model to construct LF prevalence maps for this continent [44]. These data are mapped in Fig. 1a, and the database includes country and village names, latitude and longitude of the villages, the number of individuals examined and mf positives detected, both stratified by age, as well as key infection-related environmental variables estimated for each site. However, as the existing mf surface map provides only overall mf prevalence values interpolated over a grid rescaled to 1 km × 1 km resolution [44], and as the LF models require age-stratified infection data at every site for parameterization purposes, we needed to estimate the mf age prevalences from the overall community mf prevalence for those sites where such data were unavailable. This was done by converting the community-level overall prevalence into three representative standardized mf age prevalence curves [45], by first constructing an age structure for the community in question using the pertinent national demography profile and then estimating the mf positives in each of the constructed age classes (e.g., 1–10, 11–20, etc.) based on the shape of each of the linear, plateau, and convex mf curves likely or expected to occur in a typical endemic site (see Additional file 1: Figure S1). Note that we consider such derived site-specific age prevalence curves as representing the baseline condition in the year 2000 before large-scale mass drug administration (MDA) programs began to be implemented by the Global Programme to Eliminate Lymphatic Filariasis (GPELF) in Africa. This, however, cannot be expected to be the case for the 11 countries in West Africa (Benin, Burkina Faso, Cote d’Ivoire, Ghana, Guinea Bissau, Guinea, Mali, Niger, Senegal, Sierra Leone, and Togo) that had participated in the Onchocerciasis Control Program (OCP), which began in these countries in 1975 [46, 47]. Apart from vector control (VC), these former OCP countries also had been treated with ivermectin under the community-directed treatment with ivermectin (CDTI) program over a period of 15 years from 1988 to 2002. Such prolonged mass treatment could have significantly reduced the prevalences of LF, as indeed was the case in Sierra Leone, where LF mf prevalences across various sites dropped by rates that ranged between 80 to 100% prior to the delivery of LF MDA under the national LF control program [48]. To account for the impact of CDTI in the above 11 OCP countries, we used this information conservatively and reduced the site-specific map-extracted mf prevalences in all these countries uniformly by 75%, and all baseline model fittings were carried out using these reduced mf prevalences.

### Vector mosquito species and annual biting rates (ABRs)

Information on the presence of LF vector mosquito species in countries can be obtained from the published literature, including from WHO [49]. Since there are very sparse corresponding spatial data available on ABR (Fig. 1b), we developed a reverse engineering approach to produce model-generated data to overcome this gap. This was done by estimating sets of plausible ABR values from the ensembles of fitted parameter vectors such that the model-generated mf prevalences matched either the observed or geostatistically extracted mf prevalence in a site (see details of the Bayesian melding-based ensemble modeling approach given in Additional file 1). Comparison of such model-generated ABRs for sites where measured ABRs were available showed that we were able to reasonably recover these values using this approach (Additional file 1: Figure S2).

### Intervention data on mass drug administration (MDA)

The WHO preventive chemotherapy and transmission control (PCT) databank (http://www.who.int/neglected_diseases/preventive_chemotherapy/lf/en/) provides the following information about the transmission and control status of LF world-wide: (1) LF endemic countries given by regions; (2) the mapping status of LF prevalence in the countries of these regions provided as completed, in progress, or not started; (3) information on drug regimens applied during the years in which MDA was administered; (4) years of annual MDA with the numbers of implementation units (IUs) covered in individual years at the country level; and lastly (5) information on national program drug coverages achieved during the period 1999–2013 for all countries undergoing annual LF MDA. In Africa, as of 2013 these data show that 23 countries have completed mapping status for LF; these countries have either already implemented several rounds of annual MDA or are yet to start MDA (Additional file 1: Table S2).

An examination of the MDA data available from the PCT databank (Additional file 1: Table S2) showed that not all endemic IUs received MDA since the start of mass treatment in a given country. An analysis of the data showed a clear occurrence of three distinct phases of MDA implementation in the period 2000–2013 in terms of the numbers of IUs starting annual mass treatments in each country (Table 1). These three phases can be termed as the initial phase covering the first 1–3 years of mass treatments, the expansion phase (lasting for 4–5 years after the initial phase), and the later/saturation phase in which almost all endemic IUs in a country started implementing annual MDAs. In addition, we also found a fourth group of IUs present in all LF endemic African countries except Togo, where MDA apparently had not begun or no MDA information was available by 2014–2015. We presumed that these IUs would begin annual MDAs from 2016 in all the simulations described below.

**Table 1:** Details of annual mass drug administration (MDA) delivery in LF endemic sub-Saharan African countries

Country^a^	Vector genus^b^	MDA type	IUs (initial phase: 1–3 years)	Start year	% program drug coverage (range)	IUs (expansion phase: 4–5 years)	Start year	% program drug coverage (range)	IUs (later phase)	Start year	% program drug coverage (range)
Benin	*Anopheles*	IVM-ALB	9	2002	79.35 (77.8–80.5)	21	2004	81.23 (75.4–85.6)	25	2013	100
Burkina Faso	*Anopheles*	IVM-ALB	22	2001	74.43 (68.4–77.8)	59	2005	80.3 (77.2–81.8)	57	2013	71.4
Cameroon	*Anopheles*	IVM-ALB	12	2008	75.95 (74.6–77.3)	84	2010	73.9 (69.8–78.0)	123	2012	80.35 (79.9–80.8)
Comoros	*Culex*	DEC-ALB	2	2001	73.2 (56.9–85.7)	–	–	–	–	–	–
Congo	*Anopheles*	IVM-ALB	–	–	–	–	–	–	5	2013	92.8
Cote d’Ivoire	*Anopheles*	IVM-ALB	3	2009	78.9	13	2010	31.5	5	2013	70
Egypt	*Culex*	DEC-ALB	28	2000	93.92 (91.0–96.7)	–	–	–	–	–	–
Ethiopia	*Anopheles*	IVM-ALB	5	2009	77.53 (72.7–81.7)	–	–	–	27	2012	76.4 (74.2–78.6)
Ghana	*Anopheles*	IVM-ALB	22	2000	62.28 (23.9–74.1)	66	2005	72.15 (63.9–75.2)	91	2013	76.8
Guinea Bissau	*Anopheles*	IVM-ALB	–	–	–	–	–	–	33	2011	71.7 (69.4–74.0)
Kenya	*Anopheles* and *Culex*	DEC-ALB	1	2002	81.20	3	2003	71.5 (62.7–79.5)	9	2011	58.3
Liberia	*Anopheles*	IVM-ALB	–	–	–	–	–	–	13	2012	90.7 (81.1–100)
Madagascar	*Anopheles*	DEC-ALB	3	2005	81.40	25	2006	76.68 (74.6–79.5)	39	2010	66.53 (66.3–66.9)
Malawi	*Anopheles* and *Culex*	IVM-ALB	9	2008	80.50	–	–	–	26	2009	81.68 (79.8–83.0)
Mali	*Anopheles*	IVM-ALB	9	2005	80.70 (78.2–83.2)	46	2007	78.75 (61.6–85.1)	45	2013	82.10
Mozambique	*Culex*	IVM-ALB	18	2009	71.81	51	2010	77.85 (74.4–81.3)	94	2013	85
Niger	*Anopheles*	IVM-ALB	9	2007	72.20	20	2008	65.60 (58.7–72.5)	30	2010	69.28 (63.7–72.7)
Nigeria	*Anopheles*	IVM-ALB	14	2000	41.53 (32.3–53.7)	31	2003	72.63 (64.2–77.1)	135	2009	58.08 (48.9–73.6)
Senegal	*Anopheles*	IVM-ALB	7	2007	73.60 (66.8–78.7)	–	–	–	13	2013	49.3
Sierra Leone	*Anopheles*	IVM-ALB	6	2007	61.30	13	2008	72.1 (70.1–74.1)	14	2010	79.5 (77.7–80.6)
Sudan	*Culex*	IVM-ALB	–	–	–	–	–	–	2	2013	87.8
Tanzania	*Anopheles* and *Culex*	IVM-ALB	17	2000	76.10 (59.1–100.0)	25	2003	70.54 (60.5–79.9)	84	2011	73.03 (65.8–77.1)
Togo	*Anopheles*	IVM-ALB	7	2000	67.20	–	–	–	–	–	–
Uganda	*Anopheles*	IVM-ALB	6	2002	66.80 (53.5–76.0)	38	2007	67.5 (62.4–72.4)	41	2012	66.9 (66.8–67.0)

MDA was implemented across a country in a staggered manner, effectively creating several cohorts of implementation units (IUs), namely those that began to receive MDA in the initial phase, the expansion phase, or the later phase

^a^The following countries not listed in the table had not yet started MDA as of 2015: Angola, Central African Republic, Chad, Democratic Republic of Congo, Djibouti, Equatorial Guinea, Gabon, Guinea, South Sudan, The Gambia, Zambia, and Zimbabwe

^b^Vector genus information was retrieved from WHO [49]

*IVM* ivermectin, *ALB* albendazole, *DEC* diethylcarbamazine citrate

### Information on vector control (VC) methods and coverages

Information on VC methods for African countries was assembled from the country-specific Demographic and Health Surveys (DHS) sites (www.dhsprogram.com) as well as from WHO [49]. In the majority of countries, household usage of bed nets, mainly insecticide-treated nets (ITNs), has been the mainstay of the approach to control mosquitoes primarily under the Roll Back Malaria program (www.rollbackmalaria.org). We therefore used the ITN coverage data available for the period 2000–2012 for countries in Africa from the MAP database (Malaria Atlas Project, Oxford: www.map.ox.ac.uk) in order to derive estimates of VC efforts in this study. These data are available at the administrative level 1 (i.e., region/province level, Admin1 level hereafter), but tend to exhibit missing information for a few administrative units in one or more years, which complicated the calculations of annual VC coverage rates. In order to overcome this problem, we first created smooth maps of VC coverage for 2000–2012 using the available Admin1 data via simple kriging carried out using ArcGIS. Then, the national-level annual VC coverage values were extracted by simple averaging of the smoothed values occurring within each country boundary. These extracted annual coverages are shown in Fig. 1c and Additional file 1: Figure S3. These data showed the occurrence of high temporal variability (i.e*.*, they describe sudden jumps from a low to very high value or vice versa in the coverage values) between years within a country. We smoothed this temporal variability in the country-level data by modeling trends in the annual values observed in each country. As shown in Additional file 1: Figure S3, this predictive model was able to capture the general trend in the VC coverage levels in the studied countries for the period 2000–2012. Thus, in the present analysis, we used the smoothed annual coverage values for 2000–2012 and the predicted values for the future time period in all the LF intervention scenarios where VC was applicable.

### Data-driven LF models

The technical details of the LF transmission models used and the Bayesian melding approach employed to calibrate these models to local data, as well as specifics of how LF MDA and VC interventions are simulated, have been described extensively previously [21, 22, 50, 51] and are outlined in Additional file 1. Here, our focus is on the coupling of this data-driven modeling framework to input data assembled at local settings (here at the village level) as a means for better capturing the effects of local spatial heterogeneity in LF transmission dynamics when making predictions of the impacts of applied interventions at higher spatial scales. We describe the algorithm that used this coupling for facilitating modeling of the outcomes of the various currently applied or proposed LF intervention strategies within each study country below.

### A spatially hierarchical algorithm for modeling LF interventions

Our hierarchical algorithm for discovering and using local LF models for simulating the effects of interventions within each country comprised the following steps:

1. **Extracting and retaining a sample of overall mf prevalence values**: This was done by first constructing an Africa-wide village point map using data on populated places (city, village) within the continent contained within the GeoNames geographical database (http://www.geonames.org). These points were then overlaid on the smooth LF prevalence map of Africa [44], and mf prevalence values for all population points were extracted. This procedure resulted in a total of 342,386 mf data points for 35 of the LF endemic countries in Africa. This total does not include data points for Comoros, as there is a lack of a smooth LF surface map for the country. For this country, we used data from the LF literature (see [44] for the source references) to first estimate the maximum and minimum mf prevalences observed within the country and then used the Latin hypercube sampling technique to sample a set of 100 values within this range for use in the modeling analysis.
2. **Proportionate sampling of 10% representative mf data points**: In order to reduce the computational time which would be needed to calibrate and run models for all the extracted data points, we performed the simulations described below using a representative subsample comprising 10% of the total extracted data points. This subsample was obtained using a proportionate sampling technique to maintain the shape of the distribution of the original extracted overall mf prevalence values for a given country. This proportionate sampling was achieved in two substeps:
  - We first fitted a nonparametric distribution to the extracted overall mf prevalence data points for each of the LF endemic countries. We used the built-in *fitdist* method (available in Matlab 2014b) to fit an empirical distribution to the overall mf prevalence data with the *Epanechnikov* kernel option to perform this operation.
  - We then generated a set of random values (a total of N_C_ points) of overall mf prevalence from the fitted distribution for a country. Note here that N_C_ is set to comprise 10% of the total extracted mf points for a given country C. This draw of random values was accomplished using an empirical cumulative distribution function (CDF)-based approach in Matlab while maintaining the proportionality of the sample sizes in all of the intervals of the original data. In each case, the CDF was constructed from the nonparametric probability distribution function used in the previous step. These substeps are illustrated in Additional file 1: Figure S4.
3. **Fitting models to overall baseline mf prevalence**: There are five substeps involved here:
  - Select one mf value from the mf prevalence data sample obtained in step 2(b).
  - Select *N* (=2500) parameter vectors. Along with each sampled parameter vector, an ABR value is also needed for simulating endemic conditions. The *fzero* function in Matlab was used to identify the value of ABR which, along with the selected parameter vector, results in a model-predicted overall prevalence that equals the selected mf value from step 3(a) within a tolerance of 0.1%. The minimum and maximum values of ABR over which the function operated were 0 and 50,000 bites/person/year, respectively. This step generates a set of *N* model outputs for the selected mf value.
  - Construct the three mf age profiles (namely, *plateau*, *linear*, and *convex* age curves) for each picked mf value in step 3(a) using the actual population demographics of the country and the corresponding mf age prevalence curve (see Additional file 1: Figure S1).
  - Use the sample importance resampling (SIR) algorithm with replacement to select *S* (=200) best-fitting parameter vectors out of the sampled *N* (=2500) for each of the age profiles constructed in step 3(c). This step provides a set of 3S (=3*200) parameter vectors that have the highest likelihoods for describing each of three constructed mf age profiles.
  - Repeat steps 3(a) through 3(d) for all the subsampled mf data points for a given country, resulting in a set of N_C_*3*200 best-fitting parameter vectors per country, where N_C_ is the size of subsampled mf data points for any given country. We note that in the case of Comoros we used only 100 mf data points, sampled using Latin hypercube sampling as described above. In addition to Comoros, the total number of extracted data points for Djibouti was only 26. In this case, all data points were included.
4. **Reduced sample of baseline best-fitting models to simulate LF intervention scenarios**: The minimum and maximum sizes of the 10% sub-sampled mf data points across 34 countries were, respectively, 146 (The Gambia) and 4470 (Nigeria), which meant that a total of 10,587,600 (=N_C_*3*200) best-fitting parameter vectors were available for further modeling analyses (e.g., simulating the impact of LF intervention under different scenarios). As running intervention simulations for all these selected parameter vectors proved computationally intensive, we further reduced the size of the best-fitting parameter vectors by model selection using a cluster analysis as follows:
  - Perform a cluster analysis (we used the built-in *kmeans* function with an option of three clusters in Matlab 2014b; this option was used because of the presence of three mf age prevalence curves in the model outputs) of the best-fitting models to assess the contribution of the models describing each age curve to the full ensemble of model-predicted mf age curves.
  - Sample 10% of the best parameter vectors proportionately from each cluster based on cluster size to produce an ensemble of reduced size. This is illustrated in Additional file 1: Figure S5.
5. **Modeling of questions of interest:** Perform all modeling analyses on the reduced ensemble from 4(b) to address the variables and questions of interest (such as threshold biting rates and mf/worm breakpoints for the derivation of values signifying different elimination probabilities and running simulations of various LF intervention scenarios). Details of all equations and parameters related to these modeling activities are described in Additional file 1.
6. **Assessment of LF elimination by 2020 and simulating impacts of remedial measures**: Interventions were modeled in all countries from baseline to the year 2020 to assess whether elimination will be achieved by the target year. For this evaluation, all interventions were simulated based on actual MDA and VC coverages until 2015, after which further simulations were carried out using the most recently recorded MDA coverage and 58.78% VC coverage. For those countries which had not started MDA as of 2015, MDA and VC were assumed to have begun in 2016. We considered a country to have achieved LF elimination if the mf prevalence from each model parameter vector selected (as per step 4(b)) for modeling from that country crossed its own site-specific 95% elimination probability threshold [22, 50, 52].

In addition to assessing the achievement of elimination under the current strategy, several remedial strategies were also modeled. All remedial strategies were simulated beginning in 2016 to assess when elimination can be achieved under these improved strategies (via increased coverage, frequency of drug treatment, or by switching to a new combination drug regimen (Table 2)). Analysis of remedial strategies was not undertaken for those countries that were predicted to have achieved LF elimination under their current approaches. Table 2 outlines all intervention strategies, current and remedial with corresponding coverages, used in this work.

**Table 2:** Modeled intervention scenarios

Scenarios	MDA type	MDA coverage	VC coverage
MDA1	Annual	CC	CC
MDA2	Annual	CC	80%
MDA3	Annual	80%	CC
MDA4	Annual	80%	80%
Bi-MDA1	Biannual	CC	CC
Bi-MDA2	Biannual	CC	80%
Bi-MDA3	Biannual	80%	CC
Bi-MDA4	Biannual	80%	80%
IDA1	Annual	CC	CC
IDA2	Annual	CC	80%
IDA3	Annual	80%	CC
IDA4	Annual	80%	80%

MDA-based LF interventions were simulated for the following 12 intervention scenarios where MDA represents the standard two-drug regimen (diethylcarbamazine-albendazole) delivered annually, Bi-MDA represents the standard two-drug regimen delivered biannually, and IDA represents a triple-drug regimen (ivermectin-diethylcarbamazine-albendazole) delivered annually. Each type of MDA is combined with vector control (VC) at either current (CC) or enhanced (80%) coverage

### Scientific workflow for implementing the modeling framework

**Fig. 2:**
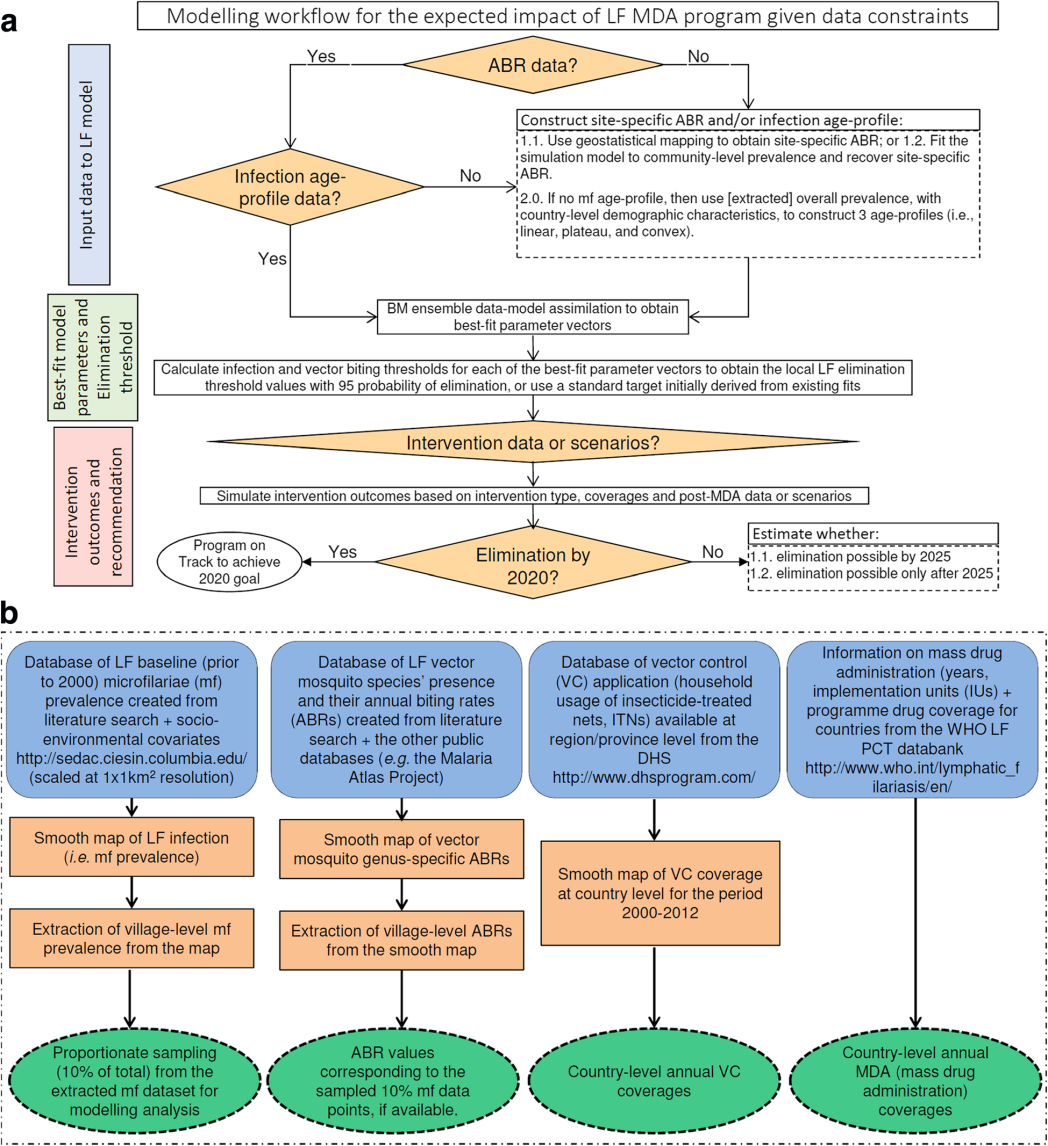
Modeling workflow and data inputs for the modeling analysis obtained from literature searches and publicly available databases. **a** The top schematic depicts the hierarchical modeling steps employed to quantify the expected impacts of MDA-based intervention programs to predict timelines to achieve the elimination of lymphatic filariasis (*LF*) from an endemic country. **b** The bottom diagram shows the steps, mainly involving geostatistical mapping, on how the data inputs required to initialize the LF model were obtained from several databases, either publicly available or created for this study from literature searches. Note that although we began examining the development of an ABR smooth map, the sparseness of ABR data precluded their reliable construction for use in this study. We instead used model fits to mf prevalence in a site to estimate ABR input values (see Methods)

We implemented the scientific workflow depicted schematically in Fig. 2 to perform the spatially hierarchical modeling exercises reported here. Figure 2a describes the sequence of steps involved in calibrating the LF model to local conditions, simulating the impacts of interventions, and assessing prospects for elimination. Figure 2b defines the data sources and pipelines we used for assembly, integration, and transformation of data into the information required by the LF transmission models as inputs. Briefly, we began the modeling process by determining whether key model input data (e.g., baseline ABR: the number of bites, on average, a person receives per year) and/or LF infection (mf age profile) were directly available from existing databases or needed to be derived for a site. Once these input data were either collated or estimated, the relevant LF model was parameterized for individual localities using the Bayesian melding algorithm, and the quantities of interest for modeling the prospects of parasite elimination, viz*.,* the local infection and vector biting thresholds, were calculated via the fitted models. Intervention modeling was then undertaken using the available or estimated data on MDA coverage and duration and on supplemental VC, for predicting the timelines to crossing the model-derived elimination thresholds in each site. Note that if intervention data are not available, simulations can also be done using hypothetical intervention scenarios at this stage. This exercise allowed us to determine if implemented and/or hypothesized interventions will lead to parasite elimination by a set target date. In cases where the modeling analysis indicated that interventions (actual or hypothesized) are unlikely to achieve elimination by a given target year for a site (e.g., by 2020), simulations for estimating the required remedial measures (e.g., increasing MDA coverage and frequency, switching to annual ivermectin-diethylcarbamazine-albendazole (IDA), including or increasing supplementary background VC using long-lasting insecticidal nets (LLINs)) to meet the goal of elimination for various target end dates were undertaken, and the outputs evaluated.

## Results

### Learning ensembles of local LF transmission models

**Fig. 3:**
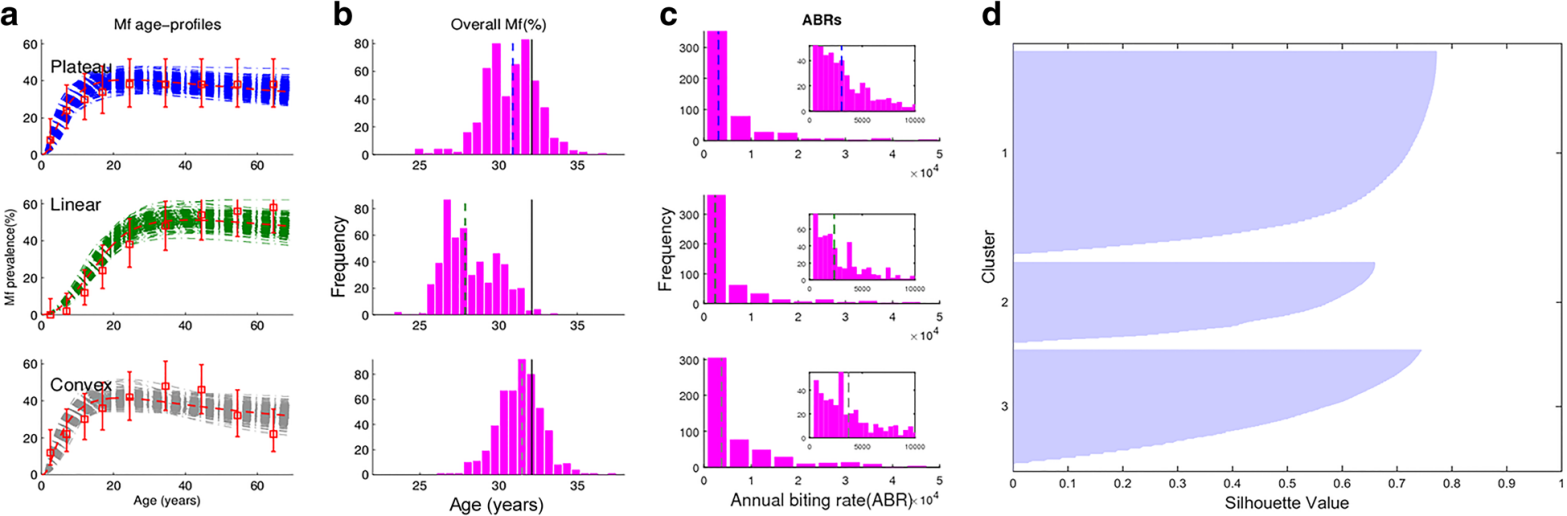
Learning ensembles of local LF transmission models. **a** An example of baseline fits to three age profiles (*boxes* depicting mean prevalences predicted in each respective age class with the *vertical lines* showing the 95% confidence intervals of these means) derived for a site in the country of Senegal with overall mf prevalence of 32.1%. **b** The corresponding predictions of overall mf prevalence are shown in a histogram where the *solid vertical line* represents the known overall mf value and the *dashed vertical line* represents the median of the predicted distribution. **c** The predicted distribution of baseline annual biting rates (*ABRs*) from the fits to each age-infection profile is shown with the median indicated by a *vertical dashed line*. The *inset plot* zooms in on the distributions in the lower ABR region. **d** A cluster plot showing the relative contributions of each age-infection profile to the pooled fits across all modeled sites in Senegal. For this country, the plateau-type age-infection model contributed most to infection for the sampled sites, followed in importance by the convex and the linear age models

Figure 3a shows an example of the fits of the anopheline LF model (broken bars) to the three characteristic age profiles (boxes depicting mean prevalences predicted in each respective age class with the vertical lines showing the 95% confidence intervals of these means) of expected mf prevalences derived for a site in Senegal depicting an overall mf prevalence of 32.1%, as obtained using our site-specific data-driven modeling algorithm. The corresponding predictions of overall mf prevalences and ABRs from the model fits to each age profile of infection are shown in Fig. 3b and c, respectively; these results highlight not only how our modeling approach can allow estimation of LF infection patterns and ABR inputs for a site, but also how we may use the model outputs to learn about which specific type of model may capture the dynamics of parasite transmission in a particular locality. This ability is demonstrated by the results shown in Fig. 3b, which compares the overall mf prevalences predicted by the ensembles of fits obtained for each type of age curve (histogram bars) with the mf prevalence extracted for this site (solid line = 32.1%). The median predicted prevalences of the fitted models are given by the dashed vertical lines, and the distance between these values and the derived mean mf prevalence can be used to assess which of the three modeled age profiles may best give rise to the overall mf prevalence extracted for a site. The results show, for example, that while the predictions of the plateau and convex models are close to the overall mf prevalence, the median of the linear model predictions is too distant from the mean mf prevalence value, suggesting that it is a less reasonable contributor to overall infection prevalence in this Senegalese site. Figure 3d is a cluster plot showing the relative contributions of each age-infection profile to all of the site-specific overall mf prevalence data we used to discover LF models for the country of Senegal, and shows that as a whole, the plateau-type age-infection model contributed most to infection for the sampled Senegalese sites, followed in importance by the convex and the linear age prevalence models. This cluster analysis of the ensemble of age-stratified model fits to overall mf prevalences obtained for a spatially representative sample of sites in each country was used to discover the corresponding LF models in each African endemic country investigated in this study.

### Elimination thresholds

**Fig. 4:**
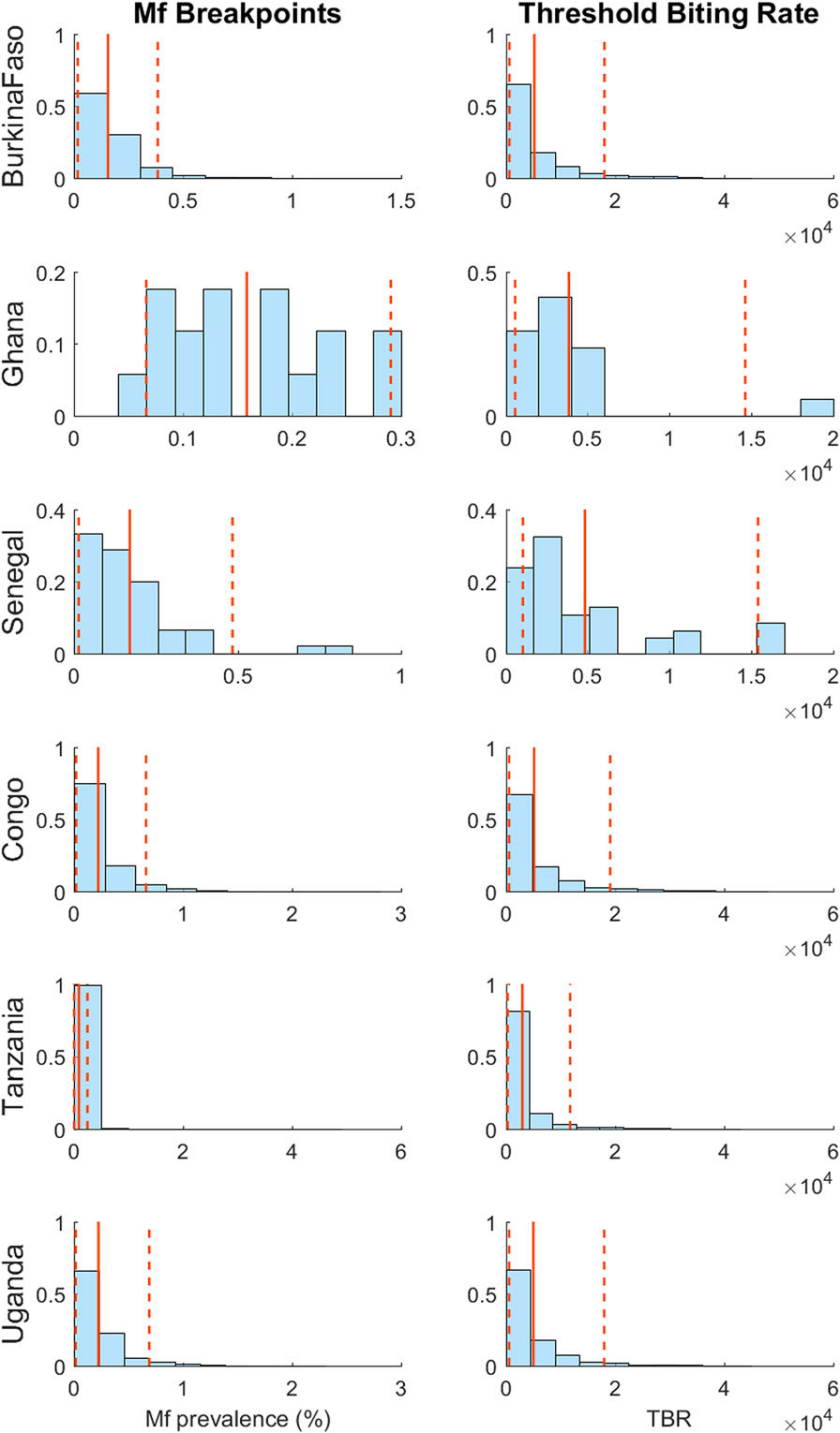
Histograms of breakpoints and threshold biting rates aggregated across modeled sites in a country. *Solid vertical lines* represent the mean value of the distribution, and *dashed vertical lines* represent the 5^th^ and 95^th^ percentile values, respectively. The histograms show that there is variation in transmission thresholds both within and between countries

We used the subset of 10% best-fitting parameter vectors selected via the cluster analysis of model fits to each selected spatially representative study site (Fig. 3d) in order to make inferences regarding the expected distribution of mf elimination thresholds within a country. Figure 4 portrays the values of mf breakpoints estimated for a selection of study countries using these best-fitting site-specific parameter vectors following the numerical method outlined in our previous work [21, 22, 50, 53]. Two features of the results depicted in Fig. 4 are immediately apparent. First, the results indicate that considerable variability in LF mf breakpoint values may occur within each country, and second, that both the distribution and mean values of these breakpoints will vary markedly between countries. Table 3 provides the mean and the 5th and 95th percentile values of the mf breakpoints obtained for each study country at both the threshold biting rate (TBR) (important as prevalence targets for assessing the impact of supplementary VC in breaking LF transmission [22, 50]) and at the prevailing ABR values in a site (important for serving as mf threshold targets for evaluating the impact of MDA [22]). The data support the impression gained from Fig. 4, and the conclusions made in our previous studies [20–22], that mf breakpoints will vary significantly between LF endemic regions, including in the present case at the country level, will be higher at TBR, and will invariably also be much lower (with 5^th^ percentile values lower by between twofold to fourfold) than the WHO-suggested threshold of 1% mf prevalence (Table 3).

**Table 3:** Aggregated country-specific mf breakpoints

Country	Mean mf breakpoint at TBR (5^th^ and 95^th^ percentiles)	Mean mf breakpoint at ABR (5^th^ and 95^th^ percentiles)
Angola	0.516 (0.0435, 2.08)	0.143 (0.015, 0.341)
Benin	0.446 (0.0609, 1.96)	0.148 (0.0289, 0.343)
Burkina Faso	0.648 (0.0436, 2.74)	0.155 (0.0163, 0.383)
Cameroon	0.7 (0.0481, 3.01)	0.188 (0.0182, 0.506)
Central African Republic	0.719 (0.045, 3.02)	0.176 (0.0172, 0.465)
Chad	0.661 (0.0435, 2.88)	0.153 (0.0165, 0.386)
Comoros	0.759 (0.0306, 3.42)	0.103 (0.00416, 0.304)
Congo	0.958 (0.0482, 3.69)	0.22 (0.02, 0.659)
Cote d’Ivoire	0.327 (0.0438, 0.861)	0.146 (0.0177, 0.337)
Democratic Republic of the Congo	0.648 (0.0435, 2.57)	0.221 (0.0159, 0.675)
Djibouti	0.632 (0.0609, 2.44)	0.178 (0.0216, 0.462)
Egypt	0.345 (0.027, 1.62)	0.118 (0.00596, 0.308)
Equatorial Guinea	0.834 (0.044, 3.33)	0.23 (0.017, 0.742)
Ethiopia	0.372 (0.0432, 1.09)	0.145 (0.0161, 0.333)
Gabon	0.951 (0.0453, 3.73)	0.238 (0.0197, 0.772)
Ghana	0.267 (0.124, 0.482)	0.158 (0.0661, 0.29)
Guinea	0.308 (0.0402, 0.806)	0.225 (0.0207, 1.1)
Guinea Bissau	0.308 (0.0402, 0.806)	0.225 (0.0207, 1.1)
Kenya	0.584 (0.0432, 2.33)	0.167 (0.0166, 0.422)
Liberia	0.777 (0.0437, 3.31)	0.189 (0.0173, 0.526)
Madagascar	0.33 (0.0325, 1.29)	0.0925 (0.00249, 0.233)
Malawi	0.578 (0.0312, 2.84)	0.0758 (0.0027, 0.218)
Mali	0.799 (0.0438, 3.34)	0.13 (0.0145, 0.302)
Mozambique	0.799 (0.0438, 3.34)	0.13 (0.0145, 0.302)
Niger	0.547 (0.0497, 1.97)	0.17 (0.0142, 0.484)
Nigeria	0.652 (0.0449, 2.2)	0.207 (0.0214, 0.758)
Senegal	0.26 (0.0319, 0.642)	0.0907 (0.0034, 0.2)
Sierra Leone	0.563 (0.0366, 2.87)	0.0929 (0.00366, 0.255)
South Sudan	0.51 (0.0363, 2.64)	0.0907 (0.00361, 0.245)
Sudan	0.901 (0.0416, 3.49)	0.188 (0.0175, 0.546)
Tanzania	0.874 (0.0386, 3.51)	0.224 (0.016, 0.689)
The Gambia	0.802 (0.0439, 3.35)	0.159 (0.0172, 0.405)
Togo	0.61 (0.0417, 2.42)	0.152 (0.0163, 0.38)
Uganda	0.516 (0.0435, 2.08)	0.143 (0.015, 0.341)
Zambia	0.446 (0.0609, 1.96)	0.148 (0.0289, 0.343)
Zimbabwe	0.648 (0.0436, 2.74)	0.155 (0.0163, 0.383)

The mean (and 5^th^ and 95^th^ percentile) mf breakpoint values by country are given under annual biting rate (*ABR*) and threshold biting rate (*TBR*) conditions. Calculations using TBR apply for interventions including supplemental VC, while those using ABR apply for interventions without VC. The relevant vector model was used for each country as indicated in Table 1. For Kenya, Malawi, and Tanzania, the results from the culicine LF model are given, as *Culex* mosquitoes represent the dominant vector in these countries. For elimination analyses, the 5^th^ percentile value represents the breakpoint value, the crossing of which corresponds to a 95% probability for achieving LF elimination

### Evaluation of elimination feasibility by year 2020 and impact of remedial measures

The principal focus of this study was to use models in conjunction with intervention data estimated or assembled at spatially representative sites in order to evaluate if currently applied interventions in LF endemic African counties will meet the goal set by WHO for achieving LF elimination on the continent by the year 2020. As described in Methods, we used a hierarchal modeling framework to undertake this analysis in order to take a fuller account of the impacts of spatial heterogeneity in transmission conditions and ultimately with respect to interventions applied across a country. Our approach was thus to first assess the prospects of eliminating LF transmission at local sites and then to aggregate this information to the IU level (this is commonly the district), and following this to the country level. In this approach, we therefore considered an IU to have accomplished the goal of LF elimination when all sites sampled within its boundary were predicted to have their infection levels reduced from baseline to below their own 95% mf prevalence elimination threshold (see Methods [22, 52]) as a result of applied interventions, and a country to have accomplished elimination at the time point when all corresponding IUs are estimated to have met the goal of elimination. Meeting the year 2020 LF elimination target was primarily assessed by inspecting if timelines predicted by local models crossed site-specific mf prevalence thresholds by 2020. Note that while we are able to estimate site-specific models and infection-related data (baseline age-stratified mf prevalences, ABRs, and mf breakpoints) to carry out the modeling analyses, intervention data (MDA start, duration and coverage, VC type and coverage) were only available at the national or IU levels. Thus, these data at a higher hierarchical level were applied uniformly to all local sites when modeling interventions in this work.

Altogether, a set of 12 intervention scenarios were investigated (Table 2). These intervention scenarios include variants of annual and biannual MDAs, and annual IDA — a triple-drug regimen comprised of standard doses of ivermectin-diethylcarbamazine-albendazole (IVM-DEC-ALB) that were recently reported to be highly efficacious against LF infection in non-onchocerciasis LF endemic areas [54]. The base intervention investigated for assessing if current MDA programs are able to bring about LF elimination by 2020 pertains to that labeled as MDA1 in Table 2, which denotes annual MDA with supplemental VC applied at the country-level coverages reported in the WHO LF PCT databank for each IU cohort in the case of MDA (Table 1), and at a coverage of 58.78% for the usage of ITNs (see Additional file 1: Figure S3). MDA2 stands for annual MDA at the most recent reported coverage in the WHO LF PCT databank, with VC coverage raised to 80% — the optimal VC coverage thought to be ideally achievable under the WHO malaria control program; MDA3 for annual MDA at 80% coverage with VC coverage at 58.78%; and finally MDA4 where annual MDA and VC both have coverage values of 80%. All four variants of MDA strategies were also considered in the case of biannual and annual IDA remedial mass treatment plans as outlined in Table 2. All remedial plans were simulated from the year 2016.

### Predictions of times to elimination by 2020 based on current MDA and background VC

**Fig. 5:**
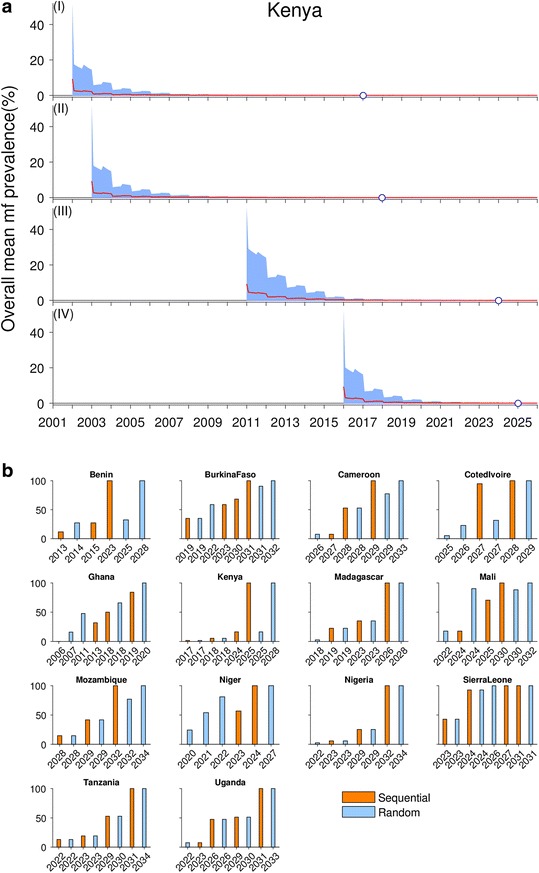
Patterns of elimination timelines of lymphatic filariasis (*LF*) by calendar years in sub-Saharan Africa. **a** An example of the effects of staggered annual MDA implementation in cohorts of implementation units (*IUs*) across a country on LF elimination trajectories, using data and model simulations for Kenya. The modeled decline in the overall mean microfilariae (*mf*) prevalence as a result of LF intervention is shown, where each cohort had a different MDA start year: 2001, 2003, 2011, and 2016, respectively. The elimination year for each cohort is shown by an *open circle* on the *x-*axis of the respective subplot. **b** Patterns in timelines to LF elimination based on annual MDAs provided to IUs either randomly or in a phased sequential manner starting with provision of treatments to the highest prevalence IUs first. The *vertical bars* show fractions of IUs in a country achieving LF elimination by calendar years. The results for sequential coverage of IUs are shown in *orange* (i.e., IUs with higher baseline endemicity receiving MDA earlier than lower endemicity IUs), and those for random coverage of IUs are shown in *blue*. The results for the random selection of IUs are more pessimistic than those for the sequential approach. Data on the years and number of IUs implementing annual MDA and drug coverages are from the WHO LF PCT databank. Future LF interventions were simulated for the current MDA and VC coverages for a given country

Figure 5 depicts the patterns of timelines to LF elimination predicted both within a country (Fig. 5a) and across a selection of representative African countries (Fig. 5b) (results shown for the MDA1 scenario). Note that site-specific mf prevalence values representing a 95% elimination probability at the TBR [22] were used as targets for signifying elimination in this exercise because of the involvement of VC in all the MDA variants investigated (Table 2). The results portrayed in Fig. 5a show firstly how the staggered delivery of annual MDA may affect the timelines to eventual LF elimination in an individual country. The simulations are based on the expected declines modeled in four cohorts of early to late phase IUs in Kenya (Table 1), which are assumed to have been treated sequentially during the phased roll-out of the MDA program in that country. The results show that, depending on the start year of MDA and base mf prevalences in these different cohorts, times to LF elimination will vary between treated IUs in a country, with delayed treatments (e.g., the late phase IU cohort beginning treatment only in year 2016) lengthening the time to LF elimination for the entire country. This result highlights the crucial importance of considering both the start years as well as baseline mf prevalences of IUs when modeling the feasibility of current MDA programs for achieving LF elimination in a country.

A problem with modeling this potential IU cohort effect on timelines to LF elimination is that the WHO PCT database currently does not provide details of the IUs in a country that underwent MDA at various start dates; only numbers of IUs that began annual MDA at different time periods are provided (Table 1). As identity of IUs is required to estimate their likely baseline mf prevalence and ABRs from our maps to serve as model inputs, this lack of information means that we currently can only attempt to quantify the likely cohort effect in a country via considering possible best- and worst-case scenarios that may apply. Here, we indicate that the best case is to consider that IUs are recruited sequentially into national programs based on their endemicity level (i.e., IUs with higher baseline mf prevalence are recruited earlier than IUs with lower endemicity). This scenario can then be contrasted with the null and worst-case scenario in which IUs are recruited into annual MDA programs randomly (i.e., the baseline endemicity of IUs does not matter in determining cohort membership).

Figure 5b compares the timelines to LF elimination by IUs under these two scenarios graphically for 14 of the 36 sub-Saharan African LF countries, while Table 4 displays the actual years by which 100% of IUs are expected to achieve LF elimination in all 36 countries under each scenario. The results depicted in Fig. 5 and given in Tables 4 and 5 highlight as expected that the numbers of rounds of annual MDA required for breaking LF transmission will be lower (by between 1 to 5 years) in the case of the sequential scenario compared to the scenario in which IUs are randomly recruited into annual MDA programs. Nonetheless, a major finding is that for both scenarios the majority of African countries, except for Egypt and Togo under randomly assigned MDAs, and Egypt, Ghana, and Togo in the case of sequential treatments, will not meet the goal of achieving LF elimination by 2020 when local extinction thresholds are used in the modeling exercise. By contrast, if the WHO threshold of 1% mf prevalence is used as a target, while 12/36 of these countries are found to be able to achieve LF elimination under the current MDA program in the random IU treatment scenario by this time point, a corresponding 24/36 or 66.6% of countries are predicted to be able to achieve LF elimination under the same MDA program in the sequential treatment scenario. These results indicate that the way in which IUs are recruited into MDA programs, their original endemicity levels, treatment coverage, and the threshold value set as targets for signifying transmission breakage will all combine to govern the expected timelines to achieving LF elimination in any given country.

**Table 4:** Comparison between random versus sequential selections of IUs for implementing delivery of annual MDAs

	Random		Sequential	
	95% EP Th	WHO 1%	95% EP Th	WHO 1%
Angola	2032	2020	2032	2020
Benin	2028	2019	2023	2018
Burkina Faso	2032	2022	2031	2018
Cameroon	2033	2023	2029	2018
Central African Republic	2033	2022	2033	2022
Chad	2033	2022	2033	2022
Comoros	2023	2010	2023	2010
Congo	2033	2022	2032	2020
Cote d’Ivoire	2029	2018	2028	2018
Democratic Republic of the Congo	2032	2021	2032	2021
Djibouti	2031	2021	2031	2021
Egypt	2020	2004	2019	2004
Equatorial Guinea	2033	2021	2033	2021
Ethiopia	2030	2020	2028	2017
Gabon	2033	2022	2033	2022
Ghana	2020	2017	2019	2017
Guinea	2031	2021	2032	2021
Guinea Bissau	2034	2018	2031	2018
Kenya	2028	2021	2025	2018
Liberia	2032	2021	2030	2018
Madagascar	2028	2021	2026	2019
Malawi	2033	2020	2030	2018
Mali	2032	2020	2030	2018
Mozambique	2034	2022	2032	2020
Niger	2027	2018	2024	2017
Nigeria	2034	2023	2032	2019
Senegal	2033	2020	2034	2019
Sierra Leone	2026	2015	2026	2015
South Sudan	2031	2020	2031	2020
Sudan	2035	2026	2034	2024
Tanzania	2034	2024	2031	2018
The Gambia	2033	2022	2033	2022
Togo	2016	2006	2016	2006
Uganda	2033	2023	2031	2019
Zambia	2033	2022	2033	2022
Zimbabwe	2033	2020	2033	2021

The elimination years shown represent the calendar year when 100% of IUs are predicted to have achieved elimination. For each method, the elimination years were calculated for the model-generated site-specific 95% elimination probability thresholds (*EP Th*) as well as for the WHO 1% threshold. The results are for the current MDA plus supplemental VC coverages (i.e., for the MDA1 intervention scenario). The sub-Saharan countries not implementing MDA as of 2015 were assumed to start MDA in 2016

**Table 5:** Number of countries predicted to achieve elimination in each period under current (MDA1) and remedial intervention strategies. See Table 2 for a description of each intervention strategy

	MDA1	MDA2	MDA3	MDA4	Bi-MDA1	Bi-MDA2	Bi-MDA3	Bi-MDA4	IDA1	IDA2	IDA3	IDA4
By 2020	3	3	3	3	4	4	4	4	4	4	4	5
2021–2025	4	1	1	1	21	23	30	31	32	32	32	31
2026–2030	8	8	11	11	11	9	2	1	–	–	–	–
After 2030	21	24	21	21	–	–	–	–	–	–	–	–

### Impact of remedial measures

**Fig. 6:**
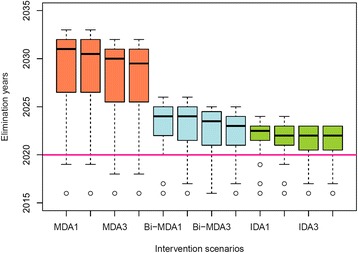
Timelines to the elimination of lymphatic filariasis (LF) from sub-Saharan Africa under various intervention strategies. Distributions of calendar years when LF elimination is predicted to be achievable in sub-Saharan Africa under the 12 considered intervention scenarios (see Table 2). These values were calculated by pooling together model-predicted country-specific LF elimination years. The country-specific LF elimination years were calculated as the year by which community-level mf prevalences in all selected spatially representative sites from a given country are predicted to be reduced below their respective 95% elimination probability thresholds (see Table 3). The *horizontal line* indicates the year 2020 — the target year set for global LF elimination. The error bars show the 2.5^th^ and 97.5^th^ percentile values



We evaluated next the effect that switching from annual MDA plus VC at current coverages from year 2016 to various remedial measures could have in accelerating the progress to LF elimination. This exercise was carried out under the sequential program expansion scenario, which we believe is a more reasonable model followed in reality compared to the random phased IU recruitment scenario, and noting that Togo, Ghana, and Egypt are expected to meet the goal of LF elimination by 2020 under the current sequential annual MDA program (Table 4). The predicted LF elimination years as a result of switching to the various currently proposed remedial drug plus VC strategies in comparison to the base annual MDA plus VC at current coverages strategy (Table 2) are summarized for all 36 countries in the form of boxplots in Fig. 6. These results show that while switching from any of the annual MDA strategies to biannual MDA and annual IDA variants will decrease the expected year of achievable LF elimination, none of the strategies will be able to eliminate LF transmission across all endemic countries on the continent by year 2020 (see also Additional file 2: Movie S1, Additional file 3: Movie S2, and Additional file 4: Movie S3). Figure 6 shows that increasing the coverages of MDA and VC from current levels to 80% will have only little impact within each MDA variant. The results also show that switching to annual IDA compared to biannual two-drug regimens will have only a small positive effect in reducing the mean year of LF elimination in Africa even in the case of the most optimal subvariant strategy, viz., providing drugs and VC at a coverage of 80%, respectively (Fig. 6).

**Additional file 2**: Movie showing impact of intervention scenario MDA1. (WMV 533 kb)

**Additional file 3**: Movie showing impact of intervention scenario Bi-MDA1. (WMV 400 kb)

**Additional file 4**: Movie showing impact of intervention scenario IDA1. (WMV 275 kb)


**Fig. 7:**
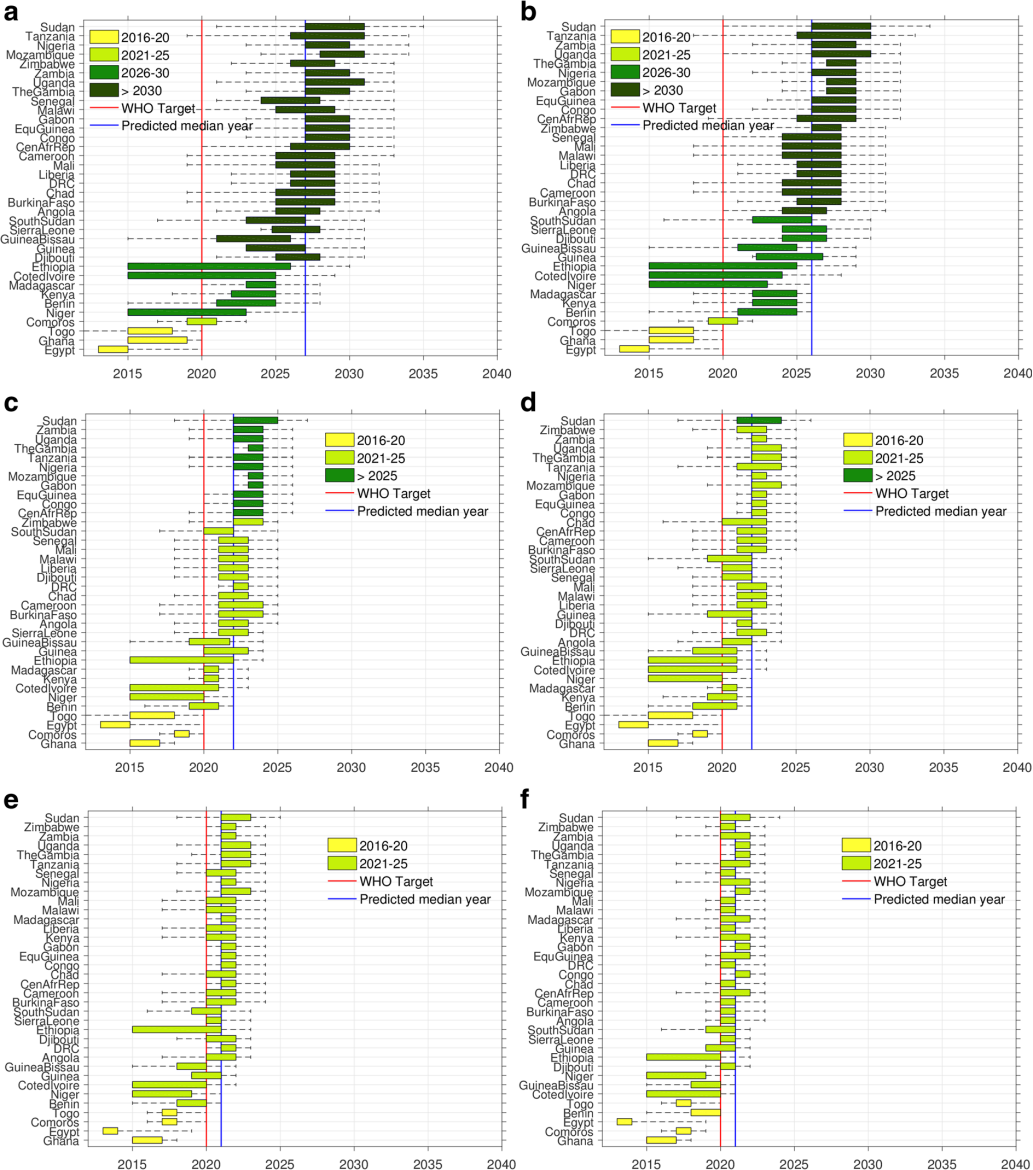
Variability in country-specific timelines to lymphatic filariasis (LF) elimination in sub-Saharan Africa. Distributions of the calendar years of LF elimination are shown for 6 out of the 12 considered remedial intervention scenarios (see Table 2). Countries are depicted in the graphs ranked by the year of elimination. Note that some countries are able to meet elimination by 2020 under their current strategy, and so would not need consideration of alternative strategies (Egypt, Ghana, and Togo). The results are shown for MDA1 and MDA4 (**a** and **b**), Bi-MDA1 and Bi-MDA4 (**c** and **d**), and IDA1 and IDA4 (**e** and **f**) (see Table 2 for descriptions). The *left panel* plots are for the current coverages of MDA and VC, while the *right panel* ones are for optimal 80% coverages for both. The widths of the boxplots denote the 25^th^ and 75^th^ percentile values of the calendar years shown on the *x*-axis. The *vertical lines* indicate the year 2020 — the target year set for global LF elimination (*red*) and the continental-wide model-predicted median elimination year for each strategy (*blue*). The whiskers show the 90% confidence interval computed using the 5^th^ and 95^th^ percentile values

Figure 7 presents the predicted elimination years due to the application of two best and worst performing variants in each mass treatment plus VC plans — the worst (the left panel plots showing the impacts of MDA1, bi-MDA1, and IDA1) and the best (the right panel plots depicting effects of MDA4, bi-MDA4, and IDA 4, respectively) by order in which individual countries in Africa may be able to eliminate LF (with the elimination year for each country under all remedial strategies shown in Additional file 1: Table S3). As shown in Fig. 7b, the results highlight that achieving LF elimination is not possible in all 36 endemic countries of sub-Saharan Africa even by 2025 using the best variant of a remedial strategy implementing annual MDA. However, if MDA delivery is switched to biannual (Fig. 7c and d), achieving LF elimination by 2025 is possible in the majority of these countries. All annual IDA-based remedial strategies (Fig. 7e and f) clearly predict more optimistic timelines to LF elimination across the continent (at the latest by 2023).

## Discussion

Using parasite transmission models for producing regional-scale intervention predictions presents a number of difficulties, which chiefly arise from the heterogeneity that underlies infection patterns across a spatial domain [17, 18, 55–58]. At the heart of these challenges is the problem of how best to scale transmission processes up from the local setting to predict phenomena at coarser hierarchical scales of space and time, particularly when inference on aggregate properties of entities of interest is based on models developed using components and processes estimated at small fine-scale levels [55–57, 59, 60]. This is compounded further by the fact that spatial variability in the biophysical and social contexts of transmission will alter the association between infection pattern and process across different endemic settings [20–22, 61, 62], and extrapolation across scales often involves transmutation, where this relationship may change qualitatively as scales are crossed [18, 63].

Here, we have sought to address the task of predicting the impact of LF interventions at the country level by employing an approach that focuses on the discovery and use of local models to take specific account of within-country spatial effects in parasite transmission and control dynamics. The approach incorporates aspects of a number of strategies suggested previously for dealing with spatial scale issues, viz., ensuring that model structure is unaltered, grain is not changed, uniqueness of location effects is preserved, and there is no aggregation of data [13, 18, 55]. This approach differs from recent attempts to model the regional impacts of interventions to either eliminate or control NTDs [1, 2, 4, 5], which largely constitute variations of the calibration approach to upscaling model predictions for generating aggregate results [18]. Here, a transmission model is calibrated against coarse-grained data, and models are identified that match the chosen data using various objective functions. While such methods can approximate the effects of spatial heterogeneity in system dynamics by selection of models that match various expected in-country infection ranges [4, 5], parameter estimates are still only valid within the often arbitrarily chosen data ranges, with the reliability of calibration unknown outside this range. They also do not account for the actual distribution of infection across a real landscape, the knowledge of which is required to appropriately weight the contribution of local models for forming more reliable aggregate predictions. Such aggregation exercises also assume that fine-scale spatial variation in parameter values within the aggregate does not matter; and finally, they presuppose the existence of an aggregate landscape-wide infection property that can be derived from the finer-scale system information [55]. The sum of these effects is that significant aggregation error will be introduced into any attempt aiming to represent what are in reality *n*-dimensional complex systems using less than *n* state variables [16]. Such errors will underestimate the impact of spatial heterogeneities in transmission processes across a domain of interest and thereby significantly bias estimates of mean timelines to parasite extinction in a spatial region [14, 17, 18].

By contrast, our technical solution to this upscaling problem is to design a hierarchical landscape-wide computational platform that facilitates learning ensembles of local LF transmission models from spatially observed/derived data within countries and uses their predictions for a representative sample of sites to support inference making at various aggregate scales (e.g., district, country, and continental levels (Figs. 5, 6, and 7; Tables 4 and 5)). In other words, the method relies on a reverse engineering paradigm in which locality-specific mechanistic models are identified and used for inference making via data-driven discovery methods [38]. This focus on data in the approach for local model discovery is important; it meant, on the one hand, the establishment of a systematic process (Fig. 2) for conducting the search, analysis, and integration of the required data, and, on the other, given gaps in these data, also consideration of how to best estimate the needed localized data using various methods of interpolation or prediction (see Methods). These vagaries in the type of input data, whether contributed by the limited availability of measured data at the scale of modeling (MDA coverages) or through errors in the data estimated for sample sites (by mapping (e.g., mf prevalence, VC coverage), model-based predictions (e.g., ABR values), or derivations (mf age prevalences)), mean that errors in model calibration and therefore in the precision of our predictions are inevitable [58]. While this cautions against the uncritical use of the present modeling results, it is also important to realize that this limitation in data for undertaking spatially structured modeling is partly procedural and therefore fixable. Thus, for example, given that much of the needed data for modeling LF control, particularly with regard to mf prevalence and coverages of MDA and VC, are available with the LF endemic countries undergoing MDA programs, provision of these data to modelers is currently hampered by the lack of negotiated data transfer protocols. Until this is resolved, it must be recognized by policy makers that modeling options, by necessity, will have to rely on resorting to simulating scenarios in those situations where data are lacking, just as we have done in this study with regard to comparing the outcomes of implementing MDA sequentially from highest to lowest prevalence IUs to a situation where MDAs are offered randomly to IUs. Our results will thereby perforce be approximate, with these data errors contributing to a portion of the uncertainty in our model outputs.

Nevertheless, one immediate value from using a data-driven modeling approach is highlighted by the staggered nature of the actual annual MDAs applied thus far in LF endemic countries (Table 1), indicating that substantive numbers of IUs within each country started MDA implementation between 2009 and 2013 with some countries beginning LF MDA nationally only at this time. This staggered start immediately points to the high probability that many African countries will not achieve LF elimination by 2020 using the current annual MDA and VC interventions. This conclusion is substantiated and further clarified by the simulations carried out in this study (summarized in Tables 4 and 5 and Figs. 6 and 7). The most significant of these results of urgent policy relevance is our finding that partly as a result of this staggered delivery of MDA, at best only 3/36 endemic countries will be able to meet the goal of LF elimination by 2020 if site-specific breakpoints are used as targets for signifying transmission interruption (with the majority of countries (21/36) able to achieve parasite elimination only between 2031 and 2035 (Table 5)), while if the WHO threshold of 1% mf prevalence is used and the sequential roll-out of annual MDAs is applicable, then this will increase to 24/36 countries able to achieve this target. This finding indicates that aggregate timelines to LF elimination from the application of annual MDA in a country will be a complex outcome of the spatial distribution of in-country baseline infection prevalences, implemented MDA and VC coverages, duration and nature of MDA roll-outs, and the infection breakpoint values used for determining transmission interruption, with the choice of which breakpoint values to use, whether the WHO-set 1% mf prevalence threshold or the much lower site-specific breakpoint values estimated in this study (see Fig. 4 and Table 3), playing the most critical role.

Our modeling of the impact of proposed or potential remedial measures applied from 2016 (using site-specific breakpoint values but following a sequential roll-out of interventions) to accelerate the progress to LF elimination in Africa has provided several new insights into the relative effectiveness of these interventions for achieving this goal. The first result of import is the finding that simply increasing VC coverage to 80% under existing MDA coverages will not accelerate the meeting of LF elimination at the country level (Table 5). This is unsurprising, given that insecticide bed net coverages used in the baseline simulations across the majority of IUs within the present countries were already at values as high as 60% on average; as we highlighted before [12, 64, 65], increasing VC coverages by moderate amounts when MDA coverages are already at moderately high levels will not lead to significant impacts on timelines to elimination due to the inherently greater impact of chemotherapy versus VC in reducing LF infection. By contrast, but for the same reason, switching to MDA based either on biannual drug delivery or annual IDA regimens significantly accelerated the achievement of parasite elimination in all countries. Thus, while implementing biannual MDA from 2016 will allow a majority of countries to achieve LF elimination under current drug and VC coverages by 2025 (see Table 5 and Fig. 6), increasing VC coverage to 80% along with this regimen will allow virtually all of them to meet the goal of elimination by this year. The best results were, however, obtained by all the IDA-based regimens evaluated, with achievement of parasite elimination facilitated in all countries by the year 2023. Although contingent on the pattern of MDA roll-outs, these results clearly support increasing suggestions for countries to switch to these more intensive drug regimens, where feasible, to accelerate their prospects for meeting the goal of LF elimination as rapidly as possible [66].

The estimates of durations required by the annual MDA intervention in this study are considerably longer than those anticipated by the Global Programme to Eliminate Lymphatic Filariasis (GPELF), which envisages all endemic countries to be under full geographic coverage by 2016 and post-MDA surveillance beginning in all countries by 2020 [67]. They are also significantly longer than estimates developed by a recent modeling study which projected that, in the worst-case scenario, LF elimination globally will be achieved by 2028 [4]. These discrepancies in the results between studies highlight the importance of carefully considering the methodologies and threshold targets used by various workers in deriving intervention duration estimates. Thus, while the GPELF estimates are simply based on assuming that 5 years of annual MDA will be sufficient to break transmission in all areas, the latter results were based on predictions of a deterministic model [12, 51, 68, 69] calibrated to a limited set of expected baseline prevalences within countries and which assumed an 85% MDA coverage and a target threshold of 1% mf for all areas. This use of uniform values for various intervention parameters, a weaker constraining of models to match only overall human infection data, plus the limited consideration of spatial heterogeneity in site-specific transmission dynamics clearly underlie the finding that 6–15 rounds of annual MDA would be sufficient to eliminate LF transmission in that work, compared to the significantly longer time periods we estimate here for the same 1% mf threshold (10–24 annual rounds between countries, depending on within-country heterogeneity in baseline mf prevalence and actual MDA/VC coverages and roll-out patterns achieved (Table 4)). This finding highlights how ignoring a fuller consideration of heterogeneous transmission dynamics across a spatial domain (as a result of not constraining a model by locally varying infection data) can lead to biased and overly optimistic aggregate predictions of the prospects for eliminating a spatially variable parasitic disease.

However, as with any modeling study, ours also has limitations that need to be considered when interpreting the results presented here. First, although our computational platform is designed to aid simulations of the effects of interventions based on discovery and use of local LF models calibrated to site-specific data, spatial correlation between sites was captured only approximately via a geostatistical model describing spatial variations in mf prevalence within an individual country. Thus, we assume that model structure is spatially invariable but parameter values will vary according to specific location as an explicit function of the spatial structure governing the distribution of this infection state variable across a region [33]. While the creation and use of ABR maps would have strengthened the incorporation of spatial structure in an important driving input variable too, this option was precluded by the lack of sufficient data on this variable for all study sites (see Methods), although note that model-estimated ABR data were used for calculating mf breakpoint values in each of our study sites. For the same reason, we have also not considered the impact of human migration patterns or mosquito dispersal patterns in our simulations, but we note that the spatial correlation in the mf prevalence data is likely to subsume some of these effects indirectly [70].

Our data-fit approach also depends on data availability as well as quality. Although here we have addressed errors in the mf/ABR data via model calibration to 95th percentile ranges in these data, it is clear that without a full observational model it is difficult to assess whether any lack of fit of our models is due to poor mechanistic understanding of the effects of spatial heterogeneity in transmission processes, or to problems in the calibration data (poor overall survey data quality, missing data). It is known that under this circumstance, model calibration efforts may need to be flexible and might need to examine the use of semiquantitative and qualitative pattern matching methods [71], rather than be based solely on quantitative data-fitting approaches [58].

Executing large continental-scale model discovery and simulation programs presents a further challenge associated with the handling and processing of large datasets. While we have created a plausible data management and scientific workflow system to tackle the issues of discovery, assembly, and data transformations/interpolations required to provide the input data for identifying the locally applicable LF models, we note that there is a need to automate our current approaches to speed up these data delivery and processing activities. We are currently working with computer scientists to develop a server-side infection data processing system based on using data warehouse principles and methods [27, 30, 31] to address this issue. A similar requirement for running data-intensive models across a large heterogeneous spatial domain is looking at advances in software and hardware to speed up the computational discovery and simulation process. This means not only optimizing our current Matlab codes for running on batch compute multicore systems and clusters, but also examining more flexible and faster code implementations using C, C++, or even Java [11]. Speeding up database and simulation scalability using hardware acceleration employing graphics processing units (GPUs) or similar accelerated parallel computing platforms [72] presents another current option we are investigating to overcome the high performance and memory overheads connected with our data-driven modeling approach. We expect that the effective resolution of these challenges will allow us to accomplish the next stage of the work reported here, viz., the provision of intervention simulations for decision making at the small spatial scale of the village or community.

## Conclusions

In summary, we present a spatially hierarchical, continental-scale, data-driven computational platform that allows the learning of local LF transmission models from georeferenced data assembled for collections of spatially representative sites in order to more reliably simulate the impacts of interventions in disrupting the transmission of this macroparasitic disease across large spatially heterogeneous domains. Our approach addresses a key challenge for producing regional-scale predictions of the impacts of national-level interventions, viz., how best to address and incorporate the locality-specific spatial as well as local to global scaling effects that are likely to influence parasite transmission processes and responses to interventions when predicting outcomes at coarser spatial scales. The results highlight that incorporating these effects into simulations will lengthen aggregate timelines to LF elimination in sub-Saharan African countries well beyond the year 2020 target set by WHO using current interventions, although exact durations per country will depend in a complex manner on the spatial distribution of in-country baseline infection prevalences, characteristics of how interventions are implemented practically, and the infection breakpoint values used for determining interruption of the transmission of this macroparasitic disease in local spatial settings. Our work also highlights how advances in the data sciences and computational discovery of knowledge provide important new tools for effectively modeling the transmission and control of spatially heterogeneous and ecologically complex parasitic diseases, such as LF.

## Additional files

Additional file 1: Supplementary material. (DOCX 820 kb)

## Abbreviations

ABR: Annual biting rate
ALB: Albendazole
BM: Bayesian melding
CDF: Cumulative distribution function
CDTI: Community-directed treatment with ivermectin
DEC: Diethylcarbamazine citrate
EP: Elimination probability
GPELF: Global Programme to Eliminate Lymphatic Filariasis
GPU: Graphics processing unit
ICT: Immunochromatographic card test
IDA: Ivermectin-diethylcarbamazine-albendazole
IRS: Indoor residual spray
ITN: Insecticide-treated net
IU: Implementation unit
IVM: Ivermectin
LF: Lymphatic filariasis
LLIN: Long-lasting insecticidal net
MDA: Mass drug administration
mf: Microfilariae
NTD: Neglected tropical disease
OCP: Onchocerciasis Control Program
PCT: Preventive chemotherapy and transmission control databank
SIR: Sample importance resampling
TBR: Threshold biting rate
VC: Vector control
WHO: World Health Organization
