# Supplementary material for: Continental-scale, data-driven predictive assessment of eliminating the vector-borne disease, lymphatic filariasis, in sub-Saharan Africa by 2020
Source: BMC Med. 2017 Sep 27;15:176. doi: 10.1186/s12916-017-0933-2 (PMC5615442; doi:10.1186/s12916-017-0933-2)
Supplement: Supplementary file 4 — Supplementary material. (DOCX 820 kb) [file 12916_2017_933_MOESM1_ESM.docx]

# Supplementary Material

# Part A: Model Description

## The mathematical model

Our mathematical model extends the recently developed mosquito genus-specific transmission model of LF to carry out the modeling analysis in this study [1-5]. It is also uniquely framed within a Monte Carlo based data-model assimilation (DA) framework, which facilitates the joint incorporation of information on local transmission parameters from data as well as evaluation of impacts of uncertainty and stochasticity on the values of these parameters on model outputs [1,2,6-10]. Briefly, the basic model is based on a hybrid coupled partial and ordinary differential equation system, where the population-level age-structured mean worm burden (pre-patent and patent worms denoted by P and W), mean microfilarial count (M) (i.e., the mf level in the human host modified to reflect infection detection in a 1ml blood sample) and mean acquired immunity level (I) are dynamically modeled by a set of partial differential equations over time (*t*) and age (*a*), whereas the dynamics of infective stage L3 larvae in vector hosts (mosquito populations) are modelled by an ordinary differential equation essentially reflecting the significantly faster time-scale of the infection dynamics in the vector hosts, which allows us to make the simplifying assumption that the density of infective stage larvae in the mosquito population reaches a dynamic equilibrium (denoted by L*) rapidly [1-3,11,12].

Here, the quantity$L^{*}$denotes the equilibrium density of infective L3 larvae, and $\Omega(a,t)=L^{*}\psi_{1}\psi_{2}g_{1}[I(a,t)]g_{2}[W_{T}(a,t)]$is the establishment rate of larvae in the human host moderated by effects of acquired immunity ($g_{1}[I(a,t)]$) and/or host immunosuppression ($g_{2}[W_{T}(a,t)]$) [1,2]. Note that we capture the effects of worm patency by considering that at any given time *t*, human individuals of age less than or equal to pre-patency period,$\tau,$will have no adult worms or microfilariae, i.e.,$W(a,t)=M(a,t)=0$for$a\leq\tau$, and the rate at which pre-patent worms survive to become adult worms in these individuals at$a>\tau$is given by $\zeta=\exp(-\mu_{1}\tau)$. The term$W_{T}=W+P$denotes total pre-patent and patent worms, which will govern development of immunity. The term$f(M)$describes the functional form relating the L3-stage larval uptake and development in the vector population, which is famously known to differ significantly in the two major genus of mosquito vectors implicated in LF transmission [13-16], and may be defined as [3]:

$f(M)=\left[\frac{2}{\left[1+\frac{M}{k}\left(1-\exp\left[-\frac{r}{\kappa}\right]\right)\right]^{k}}-\frac{1}{\left[1+\frac{M}{k}\left(1-\exp\left[-\frac{2r}{\kappa}\right]\right)\right]^{k}}\right]$ for mosquitoes of Anopheline genus;

$f(M)=\left(1+\frac{M}{k}\left(1-\exp\left[-\frac{r}{\kappa}\right]\right)\right)^{-k}$ for mosquitoes of Culicine genus.

In the above, $k[=k_{0}+k_{Lin}M]$is the shape parameter of the negative binomial distribution indicating that mean L3 output is dependent on the distribution of mf, typically found to be over-dispersed among hosts in a community [11,17], whereas r and$\kappa$are respectively the rate of initial increase and the maximum level of L3 larvae that develop in each vector population. The details of the derivation of these two larval uptake and development functions are given elsewhere [3]. This basic coupled immigration-death model structure as well as recent extensions have been discussed [1-3,11,12]. All model parameters, their description, and the density-dependent functions expected to modify or regulate transmission are described in SM Table 1.

**Table S1 - Model parameters and density-dependent functions**

| **Parameter**  **Symbol** | **Definition**  **(*units*)** | **Range** | **Source** |
| --- | --- | --- | --- |
| $\beta$ | Number of bites per mosquito (*per month*) | [5, 15] | [1-3,18,19] |
| $\tau$ | Pre-patency period | [7, 11] months |  |
| $\psi_{1}$ | Proportion of L3 leaving mosquito per bite | [0.12, 0.7] | [20] |
| $\psi_{2}$ | The establishment rate^1^ | [0.0000398, 0.00364] | [1-3,21] |
| $\mu$ | The worm mortality rate  (*per month*) | [0.008, 0.018] | [1-3,22-25] |
| $\alpha$ | Production rate of microfilariae per worm  (*per month*) | [0.25, 1.5] | [1-3,20] |
| $\gamma$ | The death rate of the microfilariae  (*per month*) | [0.08, 0.12] | [1-3,20,24] |
| $g$ | Proportion of mosquitoes which pick up infection when biting an infected host | [0.259, 0.481] | [1,2,26] |
| $\sigma$ | Death rate of mosquitoes  (*per month*) | [1.5, 8.5] | [1,2,21] |
| $\kappa$ | Maximum level of L3 given mf density | [3.955, 4.83] | [1,2] |
| ***c*** | Strength of acquired immunity | [0.0000003, 0.0109] | [1,2] |
| $\delta$ | Immunity waning rate (*per month*) | [0, 0.000001] | [1,2] |
| *m* | Ratio of number of vectors to hosts | $MBR/\lambda$ | Model estimated |
| *H_Lin_* | A threshold value used in$h(a)$to adjust the rate at which individuals of age *a* are bitten: linear rise from 0 at age zero to 1 at age *H_lin_* in years.$h(a)=a/H_{Lin}\text{ for }a<H_{Lin};h(a)=1\text{ for }a\geq H_{Lin}$ | [12, 360] months | [1,2,12] |
| $r$ | Gradient of mf uptake^2^ | [0.0495, 0.22] | [1,2] |
| $I_{C}$ | Strength of immunosuppression^3^ | [0.5, 5.5] | [1,2] |
| $S_{C}$ | Slope of immunosuppression function^4^  (*per worm/month*) | [0.01, 0.19] | [1,2] |
| $k_{0}$ | The basic location parameter of negative binomial distribution used in aggregation parameter  ($k=k_{0}+k_{Lin}M$) | [0.000036, 0.00077] | [1,2,27,28] |
| $k_{Lin}$ | The linear rate of increase in the aggregation parameter defined above | [0.00000024, 0.282] | [1,2,27,28] |
| ***Description of the functions used in the model*** | | | |
| **Function** | **Mathematical expression** | **Parameters** | **Source** |
| ^#^Probability that an individual is of age *a* | $\pi(a)=B_{1}\exp[-B_{2}a]$ | Human age *a* in month | [1,2,12] |
| Adult worm mating probability |  | *k* – negative binomial aggregation parameter | [1-3,29] |
| Immunity to larval establishment | $g_{1}[I(a,t)]=\frac{1}{1+cI(a,t)}$ | *c* – strength of immunity to larval establishment | [1,2] |
| Host immuno-suppression | $g_{2}[W_{T}(a,t)]=\frac{1+I_{C}S_{C}W_{T}(a,t)}{1+S_{C}W_{T}(a,t)}$ | *I_C_* – strength of immunosuppression;  *S_C_* – slope of immunosuppression | [1,2] |

^1^The proportion of L3-stage larvae infecting human hosts that survive to develop into adult worms [1].

^2^The gradient of mf uptake *r* is a measure of the initial increase in the infective L3 larvae uptake by vector as *M* increases from 0 [1,12].

^3^ The facilitated establishment rate of adult worms due to parasite-induced immunosuppression in a heavily infected human host.

^4^ The initial rate of increase by which the strength of immunosuppression is achieved as$W_{T}$increases from 0 [30].

^#^The parameters$B_{1}$and$B_{2}$are estimated from human demographic data.

### General Bayesian Melding Method

*1. Notation*

Following [8], we begin by denoting the collection of model inputs about which information is uncertain by$\Theta$. These can include model parameters and starting values of a system. We note that this collection of model inputs may represent a subset of the set of model inputs, and does not consist of those inputs that, based on expert knowledge and experience, are taken to be known or fixed in the model. We represent the collection of model outputs about which we have observed information (such as the number of mf positives from the baseline LF surveys) by$\Phi$. This collection will be a subset of all the model outputs, and can include values of a few or all state variables of the system of interest at various time-points. Note, in the case of a deterministic system, we can derive a mapping function by$M_{\Phi}$such that$\Phi=M_{\Phi}(\Theta)$, *ie.* the outputs are fully expressed in terms of the inputs. The third notation represents the quantities of policy or research interest, and is denoted by$\Psi$. These quantities of interest can be functions of either model inputs or outputs, or of both, such that:$\Psi=M_{\Psi}(\Theta,\Phi)=M_{\Psi}(\Theta,M_{\Phi}(\Theta))$, which shows that $\Psi$can be represented as a function of the inputs alone. Finally, data collected from affected populations/communities provide information about the model outputs. Collection of such data is represented by$y$.

*2. Bayesian melding*

The basic idea under this method is to combine or fuse all available information about model inputs and model outputs via Bayesian synthesis, in order to yield a Bayesian posterior distribution of the quantities of interest,$\Psi.$ The first step under the BM method is thus to translate and encode the available information about model inputs and outputs in terms of probability distributions. This can be done as follows. We represent the available information about the inputs,$\Theta,$ by a prior probability distribution$q(\Theta).$We specify a conditional probability distribution of the data *y* given the outputs$\Phi,$ and this yields a likelihood for the outputs, which can be represented as$L(\Phi)=\text{Prob}(y|\Phi).$ As an aside, in the case of the modelling of LF infection age-profiles, this likelihood can be evaluated using a binomial probability function:

$$\Pr(Y=y)=\frac{n!}{y!\left(n-y\right)!}p^{y}(1-p)^{n-y}$$

where *y* is the number of mf-positive blood samples out of the total *n* blood samples collected during the baseline survey conducted in a LF endemic site with *p* being the probability of such observation in different age-classes.

As for outputs, a conditional likelihood for the inputs is expressed as follows:$L(\Theta)=\text{Prob}(y|M_{\Phi}(\Theta)).$ As we have both a prior probability density function$(q(\Theta))$and a likelihood function$(L(\Theta))$ for the inputs, following Bayes’ theorem we can obtain a posterior distribution of the inputs given all the available information. This posterior distribution density is proportional to the prior density times the likelihood of the inputs given data, and can be expressed as:

$$\pi(\Theta)\propto q(\Theta)L(\Theta).$$

A constant of proportionality can be defined given this expression such that$\pi(\Theta)$becomes a probability density. In other words, it integrates to 1 over the joint space of the inputs with a suitable choice of proportionality constant. As the quantities of policy/research interest ($\Psi$) can be expressed in terms of the inputs, the posterior probability distribution of the inputs yields a posterior distribution of the quantities of interest, which is denoted as$\pi(\Psi)$. This posterior distribution$\pi(\Psi)$thus combines all the available information on the inputs and outputs of a system in a statistically coherent way, and therefore may provide a comprehensive basis for carrying out risk assessments and decision-making about a dynamical entity [6].

*3. Simulating the posterior distribution.*

It is clear that for a complex model and due to various mapping functions, namely$M_{\Phi}$and$M_{\Psi}$, the posterior distribution$\pi(\Theta)$will not have an analytic form. However, since these mapping functions can be evaluated via computer simulations, the use of a Monte Carlo method based on the sampling importance resampling (SIR) algorithm can approximate this posterior distribution [1,2,6,7]. This works as follows (note that some values outlined here for the general case may be adapted in the main text):

1. Draw a sample$\{\Theta_{1},\cdots,\Theta_{I}\}$of values of the inputs from the prior distribution$q(\Theta)$with $I=200000$. Note that, each element (referred to as a parameter vector in this paper) of this collection comprises of all model parameters, and, in practice, the value of$I$can vary between 50000 and 200000 [1,2,31]. In other words, this sample of $\Theta$has a set of $I$parameter vectors. The random values of the inputs can be drawn for the appropriate distributions. In our case the inputs are drawn from the uniform distribution with the extremes set by the known minimum and maximum values of the model parameters based on expert knowledge and experience. See SM Table 1 for the maximum and minimum of the model parameters.
2. Obtain the collection of the corresponding model outputs$\{\Phi_{1},\cdots,\Phi_{I}\}$with the mapping function of$\Phi_{i}=M(\Theta_{i})$as defined above. In other words, the collection of the outputs is generated by simulating the dynamic model for all elements of the input collection$\{\Theta_{1},\cdots,\Theta_{I}\}$.
3. Compute weights for each of the elements in the collection of the outputs given data using$w_{i}=L(y|\Phi_{i})$. Employing the mapping function which relates the outputs with the inputs, we thus get the weights (*ie.*,$w_{i}=L(y|M_{\Phi}(\Theta_{i}))$) for all$\Theta_{i}$.
4. Use the SIR algorithm to approximate the posterior distribution of the inputs with values$\{\Theta_{1},\cdots,\Theta_{I}\}$ by resampling them (at least, a set of$l=500$[1,2,31]) from the collection with probabilities proportional to$\{w_{1},\cdots,w_{I}\}$.
5. Use the posterior distribution of the inputs to approximate the posterior distribution of the quantities of interest. The approximated posterior distribution has values$\{\Psi_{1},\cdots,\Psi_{I}\}$ where$\Psi_{i}=M_{\Psi}(\Theta_{i},\Phi_{i})$ and probabilities proportional to$\{w_{1},\cdots,w_{I}\}.$ In practice, the posterior distribution of the quantities of interest is obtained by re-running the dynamic model of the system under investigation over the resampled set (*cf.* Step 4) of the inputs. For example, we obtain the model fits to the observed mf age-profile data by re-running the model using the posterior distribution of the inputs. Similarly, the posterior is used to calculate the infection breakpoints and/or threshold biting rates (TBRs), which are then used to calculate the timelines of LF elimination under a set of intervention scenarios as discussed below.

### Numerical stability analysis for quantifying infection breakpoints and vector biting thresholds

A previously developed numerical stability analysis procedure, based on varying initial values of *L^*^* to each of the SIR selected model parameter sets or vectors, was used to calculate the distribution of mf breakpoints, and the corresponding threshold biting rates (TBR) that may be expected in each study community [10 18], as follows. Briefly, we begin by progressively decreasing *V/H* from its original value to a threshold value *below* which the model always converges to zero mf prevalence, regardless of the values of the endemic infective larval density *L^*^*. The product of $\lambda$and this newly found *V/H* value is termed as the threshold biting rate (TBR). Once the threshold biting rate is discovered, the model at TBR will settle to either a zero (trivial attractor) or non-zero mf prevalence depending on the starting value of *L^*^*. Therefore, in the next step, while keeping all the model parameters unchanged, including the new V/H, and by starting with a very low value of *L^*^* and progressively increasing it in very small step-sizes we estimate the minimum *L^**^* *below* which the model predicts zero mf prevalence and above which the system progresses to a positive endemic infection state. Here, thus, *L^**^* represents the L3 breakpoint density in the vector population. This value can be converted to a prevalence value using the relationship:$P(Z,k)=1-(1+Z/k)^{-k}$, where *P* is the L3 infection prevalence, *Z* is the L3 density (ie., *L^**^*) and *k* is the aggregation parameter of the negative binomial distribution [32]. The mf prevalence at the *L^**^* value is termed as the worm/mf breakpoint in this study [3]. The collection of mf breakpoint and L3 breakpoint prevalences from the SIR selected parameter vectors in a site, are then used to get the LF infection extinction thresholds signifying various probabilities of elimination following the method outlined in [33]. Note, however, that here we focus on the 95% elimination probability threshold to serve as targets for the intervention simulations described below.

### Modeling intervention by mass drug administration

Intervention by MDA was modeled based on the assumption that anti-filarial treatment with a combination drug regimen acts, firstly, by killing certain fractions of the populations of adult worms and mf instantly following drug administration. These effects are incorporated into the basic model by calculating the drug-induced removal of worms and mf:

$$\left.\begin{matrix} P(a,t+dt)=(1-\omega C)P(a,t) \\ W(a,t+dt)=(1-\omega C)W(a,t) \\ M(a,t+dt)=(1-\varepsilon C)M(a,t) \end{matrix}\right\} \text{ at time }t=T_{MDA_{i}}$$

where$dt$is a short time period since the time point$T_{MDA_{i}}$when the *i*^th^ MDA was administered. The parameters$\omega$and$\varepsilon,$are drug killing efficacy rates for the two life stages of the parasite while the parameter *C* represents the drug coverage. Apart from instantaneous killing of mf, LF drugs are also thought to continue to kill the newly reproduced mf by any surviving female adult worms for a period of time$T_{p}$. We model this effect as follows:

We simulated the effects of MDA interventions by running the model with fixed-values of the three drug-related parameters (*ω*, *ε* and$T_{p}$). The values of worm and mf kill rates for the drug regimens studied here were taken from [5]. The first MDA round is implemented in the model by applying the above equations to the parameter vectors obtained from the baseline fits describing the pre-control worm (*W*) and mf (*M*) loads in each site, and subsequent interventions are simulated as periodic (yearly and six-monthly, respectively, for annual and biennial MDAs) events acting on parasite loads resulting from each sequentially applied MDA. We investigated the impact of annual and biennial MDAs on the years of mass treatment required to reduce mf prevalence from baseline to below the elimination threshold estimated for each site. In the model, the effect of drugs was modelled in the subpopulation of 5 years old and above.

**Modeling vector control by LLINs**

We modelled supplemental vector control (VC) applications in terms of the impact of long lasting insecticidal nets (LLINs) by assuming that population-level coverage of LLINs would reduce the vector biting rate to the same degree regardless of the mosquito genus present in a study site. Insecticides used in LLINs have three important effects on vector mosquitoes [34,35]: they deter mosquitoes from entering human dwellings; inhibit them from taking human blood meals; and kill them. These three effects can be combined for modelling the effect of VC applications on the prevailing ABR. Note that the VC efficacies decay over time, for example, due to wear and tear of bed nets used in the households [34,35]. We also assume that the LLINs applications in households are regularly replenished or renewed over a recommended time-period [35]. In this study, we consider this period to be of three years for LLINs. Taking into account the decay and periodic replenishment of insecticides, the impact of VC in this work is modelled by extending our previous formulation [5,12]; whereby we replace$\frac{V}{H}$in the model equation by the term:$\frac{V}{H}(1-\eta_{1}\exp[-\Lambda t]C_{VC})(1-\eta_{2}\exp[-\Lambda t]C_{VC})(1-\eta_{3}\exp[-\Lambda t]C_{VC})$, where$C_{VC}$is the mean annual coverage level in terms of the fraction/percentage of households using LLINs in a LF endemic setting. The parameters$\eta_{1},\eta_{2},\eta_{3},$respectively, denote the level of deterrence, feeding inhibition and toxicity of the insecticides used in the manufacturing of LLINs, whereas$\Lambda$is the efficacy decay rate of the insecticides. The efficacy values of these parameters vary depending on the insecticides used in LLINs [34,35]; see Table S2 in [35]. In this study, the values of three efficacy parameters (*ie.,*$\eta_{1},\eta_{2},\eta_{3}$) used are (0.2, 0.9, 0.95) for LLINs, which were obtained by averaging across the set of insecticides used in the manufacturing of LLINs for which we have the data for the three efficacy variables. The decay rate ($\Lambda$) was fixed at 0.26/year which yields the average half-life of the chemicals used in LLIN insecticides of about 3 years (*ie.* LLINs remain, at least, 50% efficacious before they are replenished with new ones). In this study, all VC results are presented for the household LLIN coverage of 80%.

### Calculating site-specific extinction probabilities

We calculated the probability that LF extinction has been achieved in each study site by first deriving the empirical inverse cumulative density functions (ICDFs) for the ensemble of mf breakpoint prevalence values obtained in each site. The site-specific probability of LF extinction was then estimated by calculating the 95^th^ percentile mf value given the derived ICDFs in each of the present sites, and evaluating the exceedance probability of crossing below this value due to the effects of interventions [33]. These calculations were carried out using the model-estimated mf breakpoint values at TBRs in each site.

# Part B: Supplementary Figures and Tables


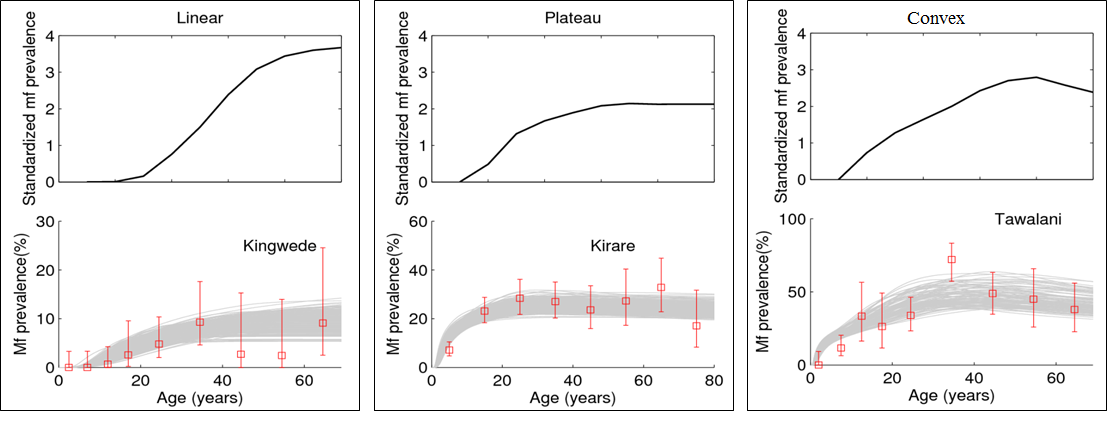


**Figure S1. An example of three representative mf age-prevalence curves that in typical communities of Africa.** The three sites shown are from Africa. The baseline data (i.e., mf age-prevalence and vector species and ABRs) shown in the bottom panel are for Kingwede in [36,37], Kirare [38], and Tawalani [39]. The top panel depicts the derived standardized curves pertaining to the 3 observed mf age-infection patterns in the panel below for use in the modelling work carried out in this paper.


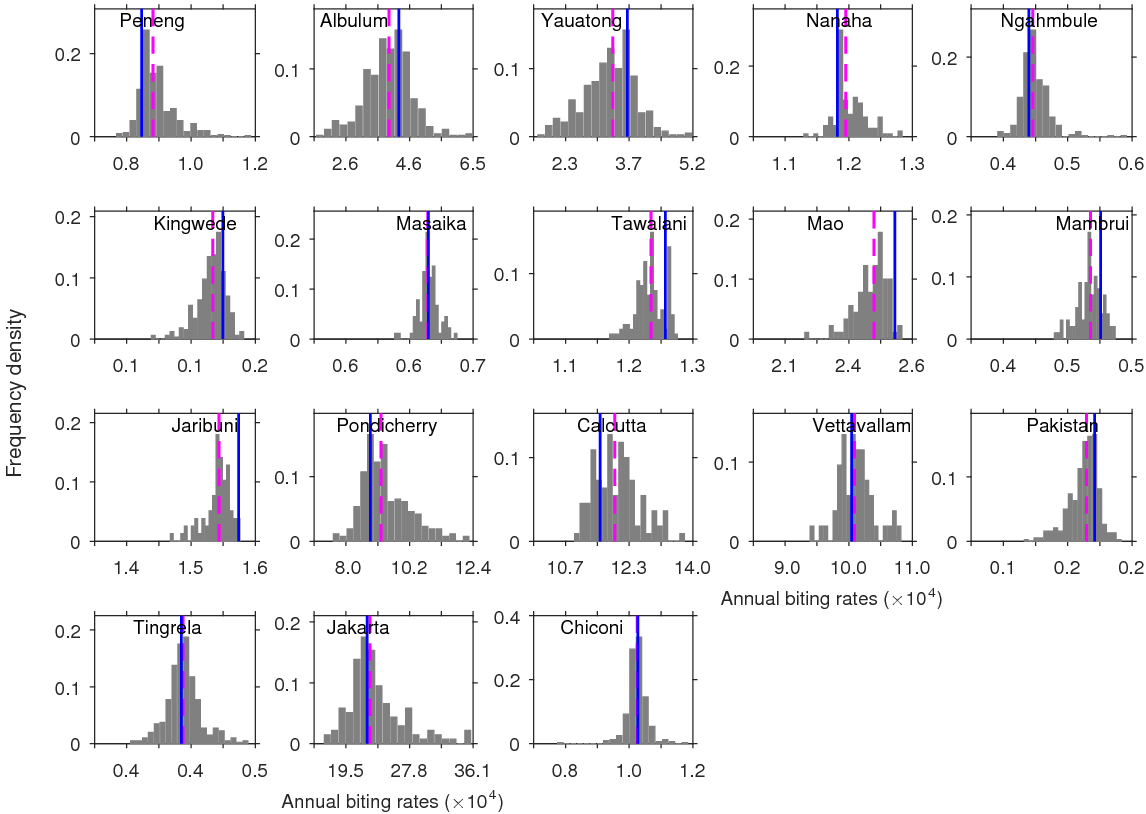


**Figure S2. Distributions of model-estimated annual biting rates (ABRs) from the baseline model fits to a number of sites from different LF endemic regions around the world.** These sites are from Papua New Guinea (Peneng, Albulum, Yauatong, Nanaha, and Ngahmbule, all with vector mosquitoes of *Anopheles* genus [1,2,40,41]), Africa (Kingwede, Mao, Mambrui [these three sites with predominant vectors mosquitoes of *Culex* genus [36,37,39]), Jaribuni, Masaika, Tawalani, Tingrela, and Chiconi (these five sites with vector mosquitoes of *Anopheles* genus [36,37,39-41]), and the South East Asia (Pondicherry, Calcutta, Vettavallam, Pakistan, and Jakarta, all with vectors mosquitoes of *Culex* genus [40,41]). The solid lines indicate the values of the observed baseline ABRs while the dashed lines the means of the distribution of the estimated ABRs.

**
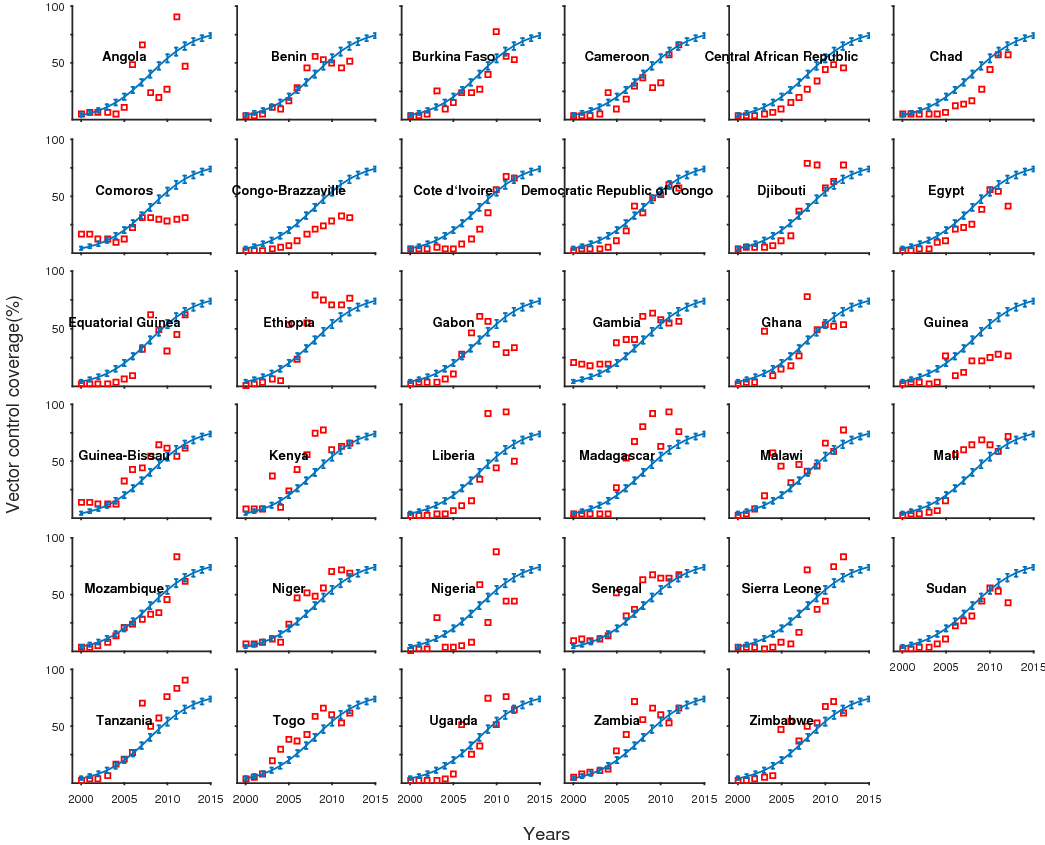
**

**Figure S3. Estimation of mean vector control (VC) coverages in African countries.** The *open* squares represent the time-varying country-specific VC coverage data. These were obtained by geostatistical modelling of ITN (insecticide treated nets) coverage data available at administrative level-1 (*i.e.* region/province level) for 35 countries shown. The *solid* (blue) line with 95% credible intervals is the fitted line to the global average of the country-specific data points. The fit was obtained using a built-in *glmfit* function in Matlab 2014b (www.mathworks.com). For years 2016 to 2050, the predicted mean values (obtained using another built-in function *glmval* in Matlab 2014b), which was saturated at the coverage level of 58.78% (not shown in these plots for the clarity sake) was used for the annual background VC coverage in the LF intervention modelling. Note that South Sudan was separated from Sudan in 2011. Hence, no VC coverage data for South Sudan is shown.

**Table S2. A summary of MDA (mass drug administration) status for LF endemic sub-Saharan African countries from the WHO LF PCT databank** (http://www.who.int/lymphatic_filariasis/en/)

| LF intervention related denominator | Number of Countries |
| --- | --- |
| Number of LF endemic countries | 37 |
| Mapping status: completed | 23 |
| Mapping status: in progress | 11 |
| Mapping status: not started | 01 |
| Type of MDA: IVM+ALB / DEC+ALB | 21/4 |
| Countries in surveillance phase | 1 |
| Countries with MDA rounds of >5 | 15 |
| Countries with MDA rounds of 2 to 5 | 8 |
| Countries with one MDA rounds | 2 |
| Countries yet to start MDA | 12 |


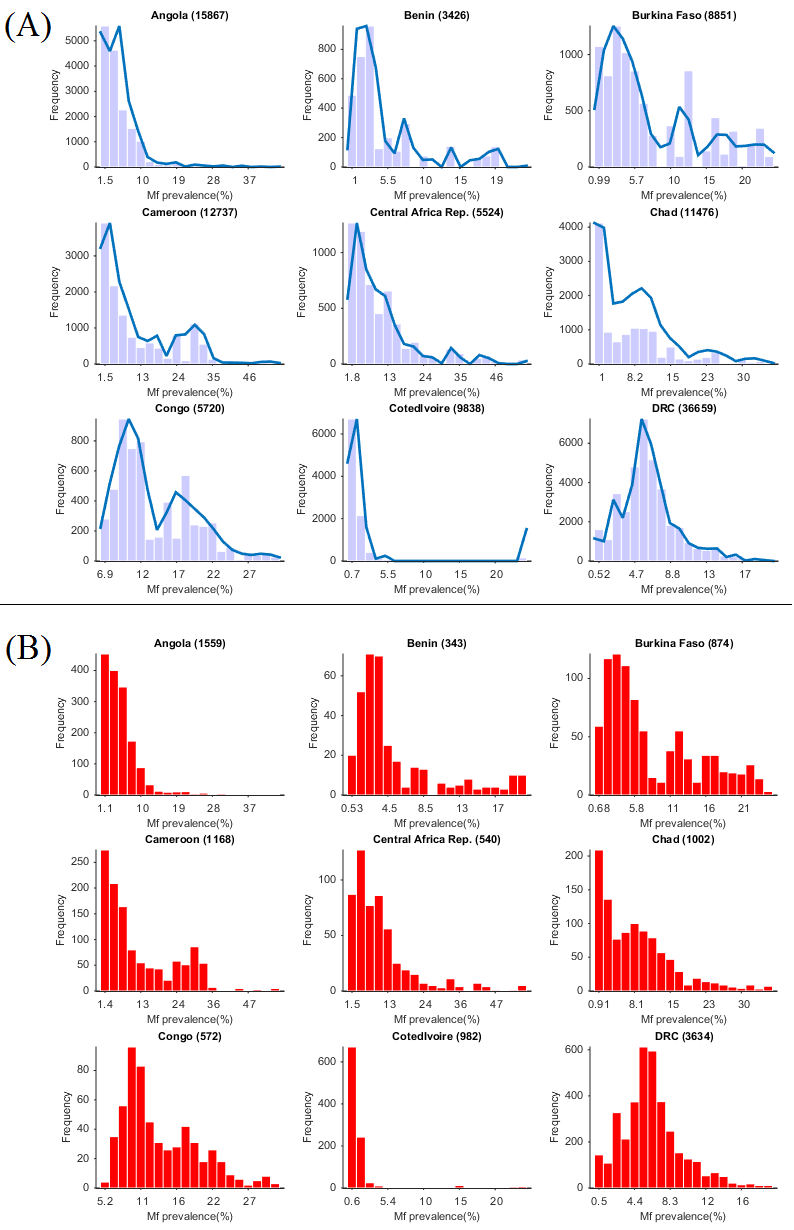


**Figure S4 - An illustration of sub-sampling of overall mf data from the distribution of the extracted data points from the smooth surface map.** In (A) a non-parametric distribution (i.e., the *solid* jig-jagged lines) is fitted to the extracted mf data for a country. In (B), the distributions of a set of random values of overall mf prevalence, which were drawn using an empirical CDF approach while maintaining the sample-size proportionality in the sample bins, are displayed. The numbers in parentheses represent the sample sizes of the extracted and sub-sampled mf data points.


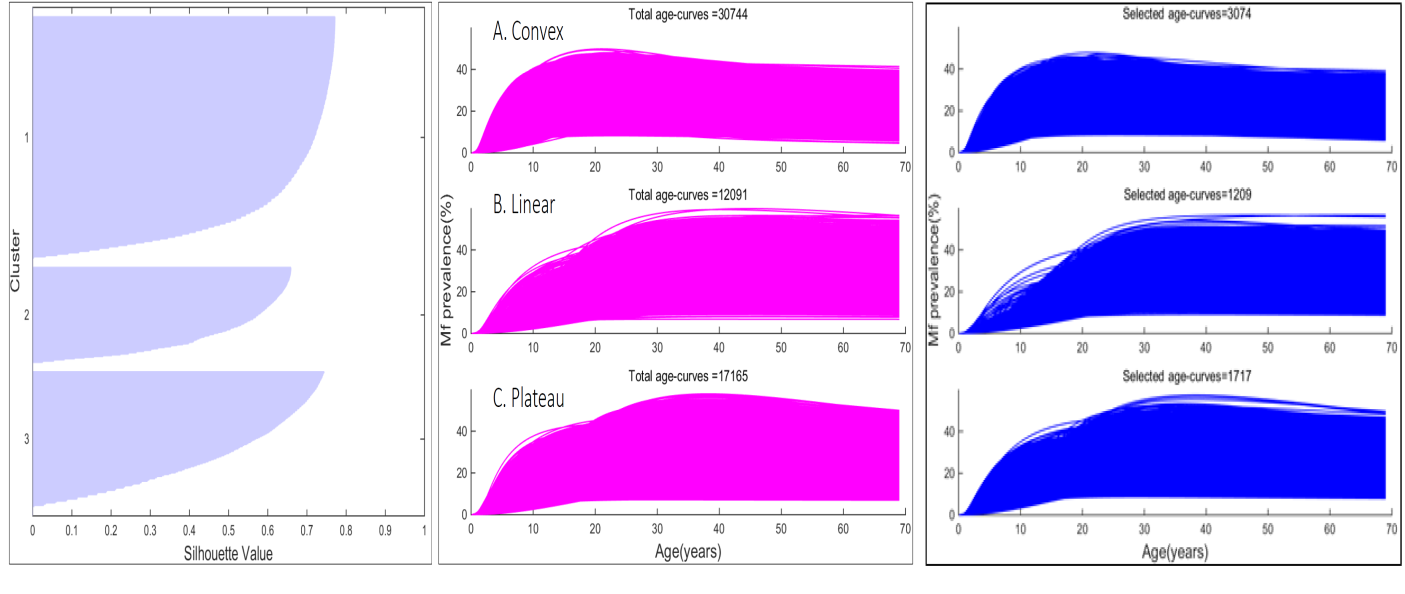


**Figure S5 - An illustration of the cluster analysis of the ensemble of model fits to a set of overall mf prevalence values for a country**. The left silhouette plot shows the contribution of each of the 3 age-curves (namely, *plateau*, *linear* and *convex*) to the ensemble fits. The middle panel shows the three model-predicted mf age-prevalence curves. The right panel shows the mf age-prevalence curve sampled proportionately at 10% of the best parameter vectors from each to perform further analyses and simulate the intervention runs.

**Table S3**. **Elimination years for 100% IUs in each country to cross their respective 95% EP thresholds under each remedial strategy.** See Table 2 in the main text for complete descriptions of the remedial strategies.

| Country | MDA2 | MDA3 | MDA4 | BiMDA1 | BiMDA2 | BiMDA3 | BiMDA4 | IDA1 | IDA2 | IDA3 | IDA4 |
| --- | --- | --- | --- | --- | --- | --- | --- | --- | --- | --- | --- |
| Angola | 2031 | 2031 | 2031 | 2025 | 2025 | 2024 | 2024 | 2023 | 2023 | 2023 | 2023 |
| Benin | 2027 | 2027 | 2026 | 2022 | 2022 | 2022 | 2022 | 2021 | 2021 | 2021 | 2020 |
| Burkina Faso | 2032 | 2031 | 2031 | 2025 | 2025 | 2025 | 2025 | 2024 | 2023 | 2023 | 2023 |
| Cameroon | 2032 | 2032 | 2031 | 2025 | 2025 | 2025 | 2025 | 2024 | 2023 | 2023 | 2023 |
| Cen Afr Rep | 2032 | 2032 | 2032 | 2026 | 2025 | 2025 | 2025 | 2024 | 2023 | 2023 | 2023 |
| Chad | 2032 | 2031 | 2031 | 2025 | 2025 | 2025 | 2025 | 2024 | 2023 | 2023 | 2023 |
| Comoros | 2022 | 2022 | 2022 | 2020 | 2020 | 2020 | 2020 | 2020 | 2019 | 2019 | 2019 |
| Congo | 2033 | 2032 | 2032 | 2026 | 2026 | 2025 | 2025 | 2024 | 2024 | 2023 | 2023 |
| Cote d’Ivoire | 2029 | 2028 | 2028 | 2023 | 2023 | 2023 | 2023 | 2022 | 2022 | 2021 | 2021 |
| DRC | 2032 | 2031 | 2031 | 2025 | 2025 | 2025 | 2024 | 2023 | 2023 | 2023 | 2023 |
| Djibouti | 2031 | 2030 | 2030 | 2025 | 2025 | 2024 | 2024 | 2023 | 2023 | 2022 | 2022 |
| Egypt | 2020 | 2020 | 2020 | 2020 | 2020 | 2020 | 2020 | 2019 | 2019 | 2019 | 2019 |
| Equatorial Guinea | 2032 | 2032 | 2032 | 2026 | 2025 | 2025 | 2025 | 2024 | 2023 | 2023 | 2023 |
| Ethiopia | 2030 | 2030 | 2029 | 2024 | 2024 | 2023 | 2023 | 2023 | 2022 | 2022 | 2022 |
| Gabon | 2033 | 2032 | 2032 | 2026 | 2026 | 2025 | 2025 | 2024 | 2024 | 2023 | 2023 |
| Ghana | 2020 | 2020 | 2020 | 2018 | 2018 | 2018 | 2018 | 2018 | 2018 | 2018 | 2018 |
| Guinea | 2030 | 2030 | 2029 | 2024 | 2024 | 2024 | 2024 | 2022 | 2022 | 2022 | 2022 |
| Guinea Bissau | 2030 | 2030 | 2029 | 2024 | 2024 | 2023 | 2023 | 2022 | 2022 | 2022 | 2021 |
| Kenya | 2027 | 2027 | 2026 | 2023 | 2022 | 2022 | 2022 | 2024 | 2024 | 2023 | 2023 |
| Liberia | 2032 | 2031 | 2031 | 2025 | 2025 | 2025 | 2024 | 2024 | 2023 | 2023 | 2023 |
| Madagascar | 2027 | 2027 | 2026 | 2023 | 2022 | 2022 | 2022 | 2024 | 2024 | 2023 | 2023 |
| Malawi | 2032 | 2031 | 2031 | 2025 | 2025 | 2025 | 2024 | 2024 | 2023 | 2023 | 2023 |
| Mali | 2032 | 2031 | 2031 | 2025 | 2025 | 2024 | 2024 | 2024 | 2023 | 2023 | 2023 |
| Mozambique | 2033 | 2033 | 2032 | 2026 | 2026 | 2025 | 2025 | 2024 | 2024 | 2024 | 2023 |
| Niger | 2027 | 2026 | 2026 | 2022 | 2022 | 2022 | 2022 | 2021 | 2021 | 2021 | 2021 |
| Nigeria | 2033 | 2032 | 2032 | 2026 | 2026 | 2025 | 2025 | 2024 | 2024 | 2024 | 2023 |
| Senegal | 2032 | 2032 | 2031 | 2025 | 2025 | 2025 | 2024 | 2024 | 2023 | 2023 | 2023 |
| Sierra Leone | 2031 | 2030 | 2030 | 2024 | 2024 | 2024 | 2024 | 2023 | 2023 | 2022 | 2022 |
| South Sudan | 2031 | 2030 | 2030 | 2025 | 2024 | 2024 | 2024 | 2023 | 2023 | 2022 | 2022 |
| Sudan | 2035 | 2034 | 2034 | 2027 | 2027 | 2026 | 2026 | 2025 | 2025 | 2024 | 2024 |
| Tanzania | 2034 | 2033 | 2033 | 2026 | 2026 | 2026 | 2025 | 2024 | 2024 | 2024 | 2023 |
| The Gambia | 2033 | 2032 | 2032 | 2026 | 2026 | 2025 | 2025 | 2024 | 2024 | 2023 | 2023 |
| Togo | 2020 | 2020 | 2020 | 2020 | 2020 | 2020 | 2020 | 2020 | 2020 | 2020 | 2020 |
| Uganda | 2033 | 2032 | 2032 | 2026 | 2026 | 2025 | 2025 | 2024 | 2024 | 2023 | 2023 |
| Zambia | 2033 | 2032 | 2032 | 2026 | 2026 | 2025 | 2025 | 2024 | 2024 | 2023 | 2023 |
| Zimbabwe | 2032 | 2032 | 2031 | 2025 | 2025 | 2025 | 2025 | 2024 | 2023 | 2023 | 2023 |

**SM Movies: Maps showing predicted timelines to lymphatic filariasis elimination on Sub-saharan Africa by country for the MDA1, Bi-MDA1 and IDA1 scenarios described in the text.**

**References**

1. Gambhir M, Bockarie M, Tisch D, Kazura J, Remais J, Spear R, et al. Geographic and ecologic heterogeneity in elimination thresholds for the major vector-borne helminthic disease, lymphatic filariasis. . 2010;8: 1.

2. Singh BK and Bockarie MJ and Gambhir M and Siba PM and Tisch DJ and Kazura J and others. Sequential Modelling of the Effects of Mass Drug Treatments on Anopheline-Mediated Lymphatic Filariasis Infection in Papua New Guinea: PLoS One; 2013.

3. Gambhir M, Michael E. Complex ecological dynamics and eradicability of the vector borne macroparasitic disease, lymphatic filariasis. . 2008;3: e2874.

4. Michael E, Malecela-Lazaro MN, Kabali C, Snow LC, Kazura JW. Mathematical models and lymphatic filariasis control: endpoints and optimal interventions. Trends Parasitol. 2006;22: 226-233.

5. Michael E, Malecela-Lazaro MN, Simonsen PE, Pedersen EM, Barker G, Kumar A, et al. Mathematical modelling and the control of lymphatic filariasis. . 2004;4: 223-234.

6. Raftery AE, Givens GH, Zeh JE. Inference from a deterministic population dynamics model for bowhead whales. . 1995;90: 402-416.

7. Poole D, Raftery AE. Inference for deterministic simulation models: the Bayesian melding approach. . 2000;95: 1244-1255.

8. Ševčíková H, Raftery AE, Waddell PA. Assessing uncertainty in urban simulations using Bayesian melding. . 2007;41: 652-669.

9. Alkema L, Raftery AE, Brown T. Bayesian melding for estimating uncertainty in national HIV prevalence estimates. Sex Transm Infect. 2008;84 Suppl 1: i11-i16.

10. Raftery AE, Bao L. Estimating and projecting trends in HIV/AIDS generalized epidemics using incremental mixture importance sampling. Biometrics. 2010;66: 1162-1173.

11. Chan MS, Srividya A, Norman RA, Pani SP, Ramaiah KD, Vanamail P, et al. Epifil: a dynamic model of infection and disease in lymphatic filariasis. Am J Trop Med Hyg. 1998;59: 606-614.

12. Norman R, Chan M, Srividya A, Pani S, Ramaiah KD, Vanamail P, et al. EPIFIL: the development of an age-structured model for describing the transmission dynamics and control of lymphatic filariasis. Epidemiol Infect. 2000;124: 529-541.

13. Southgate B, Bryan JH. Factors affecting transmission of Wuchereria bancrofti by anopheline mosquitoes. 4. Facilitation, limitation, proportionality and their epidemiological significance. Trans R Soc Trop Med Hyg. 1992;86: 523-530.

14. Pichon G. Limitation and facilitation in the vectors and other aspects of the dynamics of fi filarial transmission: the need for vector control against Anopheles-transmitted fi filariasis. . 2002;96: S143-S152.

15. Snow L, Michael E. Transmission dynamics of lymphatic filariasis: density‐dependence in the uptake of Wuchereria bancrofti microfilariae by vector mosquitoes. Med Vet Entomol. 2002;16: 409-423.

16. Snow L, Bockarie M, Michael E. Transmission dynamics of lymphatic filariasis: vector‐specific density dependence in the development of Wuchereria bancrofti infective larvae in mosquitoes. Med Vet Entomol. 2006;20: 261-272.

17. Michael E, Simonsen P, Malecela M, Jaoko W, Pedersen E, Mukoko D, et al. Transmission intensity and the immunoepidemiology of bancroftian filariasis in East Africa. Parasite Immunol. 2001;23: 373-388.

18. Rajagopalan P. Population dynamics of culex pipiens fatigans, the filariasis vector, in pondicherry: influence of climate and environment. . 1980;46: 745-752.

19. Subramanian S, Manoharan A, Ramaiah KD, Das PK. Rates of acquisition and loss of Wuchereria bancrofti infection in Culex quinquefasciatus. Am J Trop Med Hyg. 1994;51: 244-249.

20. Hairston NG, de Meillon B. On the inefficiency of transmission of Wuchereria bancrofti from mosquito to human host. Bull World Health Organ. 1968;38: 935-941.

21. Ho BC, Ewert A. Experimental transmission of filarial larvae in relation to feeding behaviour of the mosquito vectors. Trans R Soc Trop Med Hyg. 1967;61: 663-666.

22. Vanamail P, Subramanian S, Das PK, Pani SP, Rajagopalan PK. Estimation of fecundic life span of Wuchereria bancrofti from longitudinal study of human infection in an endemic area of Pondicherry (south India). Indian J Med Res. 1990;91: 293-297.

23. Evans DB, Gelband H, Vlassoff C. Social and economic factors and the control of lymphatic filariasis: a review. Acta Trop. 1993;53: 1-26.

24. Ottesen E, Ramachandran C. Lymphatic filariasis infection and disease: control strategies. . 1995;11: 129-130.

25. Vanamail P, Ramaiah KD, Pani SP, Das PK, Grenfell BT, Bundy DA. Estimation of the fecund life span of Wuchereria bancrofti in an endemic area. Trans R Soc Trop Med Hyg. 1996;90: 119-121.

26. Subramanian S, Krishnamoorthy K, Ramaiah K, Habbema J, Das P, Plaisier A. The relationship between microfilarial load in the human host and uptake and development of Wuchereria bancrofti microfilariae by Culex quinquefasciatus: a study under natural conditions. Parasitology. 1998;116: 243-255.

27. Subramanian S, Pani S, Das P, Rajagopalan P. Bancroftian filariasis in Pondicherry, south India: 2. Epidemiological evaluation of the effect of vector control. Epidemiol Infect. 1989;103: 693-702.

28. Das P, Manoharan A, Subramanian S, Ramaiah K, Pani S, Rajavel A, et al. Bancroftian filariasis in Pondicherry, south India–epidemiological impact of recovery of the vector population. Epidemiol Infect. 1992;108: 483-493.

29. May RM. Togetherness among schistosomes: its effects on the dynamics of the infection. Math Biosci. 1977;35: 301-343.

30. Duerr H, Dietz K, Eichner M. Determinants of the eradicability of filarial infections: a conceptual approach. Trends Parasitol. 2005;21: 88-96.

31. Brown T, Salomon JA, Alkema L, Raftery AE, Gouws E. Progress and challenges in modelling country-level HIV/AIDS epidemics: the UNAIDS Estimation and Projection Package 2007. Sex Transm Infect. 2008;84 Suppl 1: i5-i10.

32. Pedersen EM, Stolk WA, Laney SJ, Michael E. The role of monitoring mosquito infection in the Global Programme to Eliminate Lymphatic Filariasis. Trends Parasitol. 2009;25: 319-327.

33. Reimer LJ, Thomsen EK, Tisch DJ, Henry-Halldin CN, Zimmerman PA, Baea ME, et al. Insecticidal bed nets and filariasis transmission in Papua New Guinea. N Engl J Med. 2013;369: 745-753.

34. Griffin JT, Hollingsworth TD, Okell LC, Churcher TS, White M, Hinsley W, et al. Reducing Plasmodium falciparum malaria transmission in Africa: a model-based evaluation of intervention strategies. . 2010;7: e1000324.

35. Okumu FO, Moore SJ. Combining indoor residual spraying and insecticide-treated nets for malaria control in Africa: a review of possible outcomes and an outline of suggestions for the future. . 2011;10: 1.

36. Simonsen PE, Meyrowitsch DW, Jaoko WG, Malecela MN, Mukoko D, Pedersen EM, et al. Bancroftian filariasis infection, disease, and specific antibody response patterns in a high and a low endemicity community in East Africa. Am J Trop Med Hyg. 2002;66: 550-559.

37. Simonsen PE, Meyrowitsch DW, Mukoko DA, Pedersen EM, Malecela-Lazaro MN, Rwegoshora RT, et al. The effect of repeated half-yearly diethylcarbamazine mass treatment on Wuchereria bancrofti infection and transmission in two East African communities with different levels of endemicity. Am J Trop Med Hyg. 2004;70: 63-71.

38. Simonsen PE, Pedersen EM, Rwegoshora RT, Malecela MN, Derua YA, Magesa SM. Lymphatic filariasis control in Tanzania: effect of repeated mass drug administration with ivermectin and albendazole on infection and transmission. . 2010;4: e696.

39. McMahon J, Magayuka S, Kolstrup N, Mosha F, Bushrod FM, Abaru D, et al. Studies on the transmission and prevalence of bancroftian filariasis in four coastal villages of Tanzania. . 1981;75: 415-431.

40. Singh BK, Michael E. Bayesian calibration of simulation models for supporting management of the elimination of the macroparasitic disease, Lymphatic Filariasis. Parasit vectors. 2015;8: 1-26.

41. Michael E, Singh BK. Heterogeneous dynamics, robustness/fragility trade-offs, and the eradication of the macroparasitic disease, lymphatic filariasis. . 2016;14: 1.
